# The CPKs‐CBP60b positive feedback loop regulates immunity in Arabidopsis

**DOI:** 10.1111/tpj.71100

**Published:** 2026-09-08

**Authors:** Lu‐Shen Li, Lin Ma, Yun‐Xia Chen, Sha Li

**Affiliations:** ^1^ College of Agriculture and Biology Liaocheng University Liaocheng 252000 China; ^2^ Frontiers Science Center for Cell Responses, College of Life Sciences Nankai University Tian'jin 300017 China; ^3^ State Key Laboratory of Wheat Improvement, College of Life Sciences Shandong Agricultural University Tai'an 271018 China

**Keywords:** calcium‐dependent protein kinases (CPKs), Calmodulin‐binding protein 60b (CBP60b), transcription, phosphorylation, feedback regulation, plant immunity

## Abstract

Calmodulin‐binding protein 60b (CBP60b) is a pivotal transcription factor in the transcriptional reprogramming of immune responses. Although its downstream regulatory genes and associated genetic pathways have been reported, its post‐translational regulation and modifications remain unexplored. Here, we report that calcium‐dependent protein kinases CPK4/5/6/11 physically interact with CBP60b and phosphorylate its serine 568 residue, thereby enhancing its transcriptional activity. The phosphodeficient variant CBP60b^S568A^ compromises immune responses, whereas the phosphomimetic variant CBP60b^S568D^ enhances resistance against pathogens, demonstrating that ser568 is essential for the function of CBP60b. Moreover, we observed that the expression of *CPK4/5/6/11* is induced by pathogens, while this pathogen‐induced expression of *CPKs* is reduced in the *cbp60b* mutant. Through experiments including dual luciferase (LUC) reporter assays and chromatin immunoprecipitation (ChIP) assays, we further demonstrated that *CPK4*, *CPK5*, and *CPK11* are direct transcriptional targets of CBP60b. In conclusion, our results demonstrate for the first time that CBP60b is post‐translationally regulated by CPKs via phosphorylation, while the pathogen‐induced expression of *CPKs* is dependent on CBP60b. This mutual regulation establishes a positive feedback loop, which enables plants to mount a robust immune response against pathogens.

## Significance Statement

We report that CPK4/5/6/11 phosphorylate Ser568 of CBP60b to enhance its transcriptional activity and that CBP60b regulates the expression of *CPKs*. These findings reveal a CPKs‐CBP60b‐positive feedback regulatory loop that promotes plant immunity.

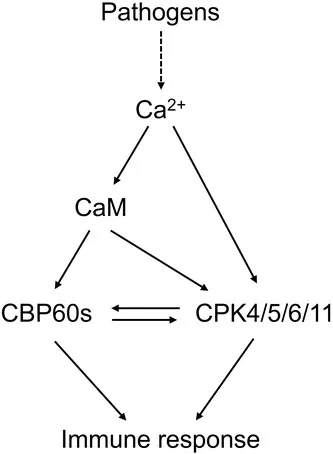

## INTRODUCTION

Plants have evolved sophisticated and intricate mechanisms throughout their evolution to sense and respond to biotic stimuli from their fluctuating environments. The plant innate immune system is initiated by cell‐surface immune pattern recognition receptors (PRRs) and intracellular immune nucleotide‐binding leucine‐rich repeat (NLR) receptors, which trigger PTI and ETI signaling cascades, respectively, thereby activating immune responses and defending against pathogen invasion (Ngou et al., 2022; Zhang et al., 2023; Zhou & Zhang, 2020). Although PRRs and NLRs possess distinct biochemical activities and are activated through relatively independent mechanisms, their downstream immune outputs are remarkably similar, including mitogen‐activated protein kinase (MAPK) cascades, reactive oxygen species (ROS) bursts, defense hormone production, Ca^2+^ influx, and transcriptional reprogramming (Jones et al., 2024; Weralupitiya et al., 2024). However, ETI‐activated immunity is often stronger and frequently induces a hypersensitive response (HR), leading to rapid localized cell death (Ngou et al., 2022; Weralupitiya et al., 2024; Zhou & Zhang, 2020). Recent studies have revealed that the previously considered relatively independent and parallel PTI and ETI pathways exhibit synergistic interactions, mutually enhancing each other to enable plants to mount sustained and robust immune responses (Yuan et al., 2023).

For a long time, Ca^2+^ influx upon pathogen infection has been recognized as a critical step in initiating immune responses (Jiang & Ding, 2023; Wang & Luan, 2024). Both the typical Ca^2+^ ion channels activated during PTI and the atypical Ca^2+^ channels formed by the assembly of pentamers upon the activation of CNLs and RNLs by pathogens in ETI lead to Ca^2+^ influx. This indicates that Ca^2+^ influx in immune responses is a common output mediated by both cell‐surface and intracellular immune receptors for immune initiation (Bjornson et al., 2021; Huang et al., 2023; Thor et al., 2020; Tian et al., 2019). However, different Ca^2+^ signals trigger distinct downstream signaling pathways with varying intensities. Therefore, decoding the spatiotemporally dynamic Ca^2+^ signals in cellular immune responses and elucidating their underlying molecular mechanisms are of significant importance (Jiang & Ding, 2023).

Among plant Ca^2+^ sensors, calcium‐dependent protein kinases (CDPKs or CPKs) are unique because they display both Ca^2+^‐sensing and protein kinase domains within a single protein. As a result, CPKs can translate the Ca^2+^ signals into phosphorylation events through stimulation of their kinase activity induced by direct Ca^2+^ binding (Jiang & Ding, 2023; Ormancey et al., 2017; Zhang et al., 2024). Two pairs of sequence‐similar CPKs, CPK4/CPK11, and CPK5/CPK6 (Yip Delormel & Boudsocq, 2019) mediate transcriptional reprogramming during immune responses in a Ca^2+^‐dependent manner (Boudsocq et al., 2010; Bredow & Monaghan, 2019; Gao et al., 2013; Zhang et al., 2024). Their downstream transcription factor substrates include WRKY8/28/48/33 and CBP60g (Gao et al., 2013; Sun et al., 2022; Zhou et al., 2020). However, the phosphorylation site identified on CBP60g is not conserved in other CBP60s proteins (Sun et al., 2022). Furthermore, CPK5‐mediated biosynthesis of SA and NHP depends on SARD1, and overexpression of *CPK5* leads to elevated *SARD1* expression (Guerra et al., 2019). It was recently reported that CPK5 promotes the degradation of CAMTA3 via phosphorylation (Liu et al., 2024). While CAMTA3 represses the expression of *SARD1* and *CBP60g*, it cannot directly bind to the *SARD1* promoter (Sun et al., 2020). This suggests that CPK5 may enhance *SARD1* expression through other transcription factor substrates.

Transcriptional reprogramming is critical in plant immune responses (Huang et al., 2023; Zhou & Zhang, 2020). Accumulating evidence demonstrates the crucial role of calmodulin‐binding protein 60 (CBP60) in immune transcriptional reprogramming (Huang et al., 2020; Kim et al., 2022; Li et al., 2024; Qin et al., 2018). As plant‐specific atypical transcription factors, the CBP60 family in Arabidopsis comprises eight members, which can be phylogenetically divided into four clades: CBP60a, CBP60b‐f, CBP60g, and SARD1 (Li et al., 2024; Zheng et al., 2021). CBP60g and SARD1 functionally redundantly regulate the expression of immune genes involved in PTI, ETI, SA, and SAR pathways (Sun et al., 2015; Wang et al., 2011) and play crucial roles in the positive feedback regulation of SA and NHP biosynthesis (Huang et al., 2020). CBP60b, a central transcription factor in immunity (Huang et al., 2021; Li et al., 2021; Li et al., 2024), not only regulates a set of immune genes overlapping with those targeted by CBP60g/SARD1 but also directly binds to the promoters of *CBP60g* and *SARD1* to modulate their expression. CBP60b may serve as a target for pathogen effectors and is surveilled by NLR proteins; its loss of function leads to autoimmunity dependent on both EDS1‐PAD4 and NDR1 signaling pathways (Huang et al., 2021; Li et al., 2021). Recent studies suggest that the CBP60b‐f clade represents the ancestral prototype of CBP60s during land plant evolution (Li et al., 2024, Zheng et al., 2021), and the CBP60b‐centered immune response mechanism is conserved in higher plants lineage (Li et al., 2024). Furthermore, the calmodulin (CaM) interacts with CBP60b‐f and CBP60g in a Ca^2+^‐dependent manner to regulate their activity during immune responses (Li et al., 2024; Reddy et al., 2002; Wang et al., 2009), indicating that their immune functions are modulated by Ca^2+^ signaling.

In this study, we observed that pathogen‐induced gene expression is reduced in the *cpk4;5;6;11* mutant, particularly for *CBP60g* and *SARD1*, which are regulated by CBP60b. This led us to hypothesize that CPKs may affect the expression of these genes through CBP60b. Consequently, we demonstrated via interaction assays that CPKs indeed interact with CBP60b and enhance its transcriptional activity by phosphorylating ser568 of CBP60b. The CBP60b^S568A^ compromises plant resistance, whereas the CBP60b^S568D^ enhances pathogen resistance to pathogens. Furthermore, we found that pathogen‐induced expression of *CPKs* is dependent on CBP60b and that *CPK4/5/11* are direct transcriptional targets of CBP60b. Collectively, our data demonstrate that CBP60b and CPKs form a feedback regulatory loop that mediates plant immune responses.

## RESULT

### CPK4/5/6/11 interact with CBP60b

**Figure 1:**
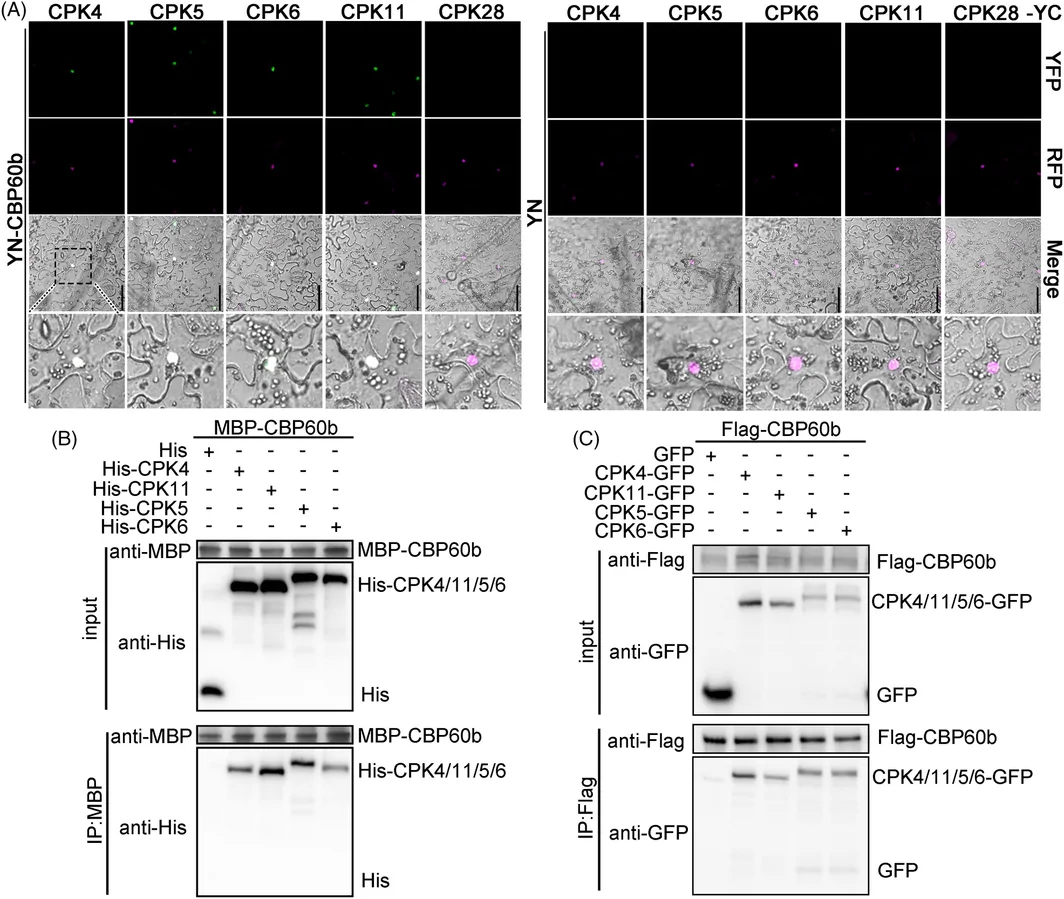
CPK4/5/6/11 physically interact with CBP60b. (A) BiFC assays showing the interaction between CPK4/11/5/6 and CBP60b. For each BiFC combination, at least 30 U1‐70K‐mRFP‐positive epidermal cells (nuclear and transformation marker) from three biological replicates were examined for eYFP signals. CPK28 as negative control. Bars = 50 μm. (B) Pull‐down showing that CPK4/11/5/6 interact with CBP60b. CPKs were fused to His; CBP60b was fused to MBP. Input and output were analyzed with anti‐His and anti‐MBP antibodies. (C) Co‐immunoprecipitation of CPK4/5/6/11 with CBP60b in planta. Flag‐CBP60b fusion protein was co‐expressed with GFP or CPK4/5/6/11‐GFP in Arabidopsis protoplasts. Protein extracts were incubated with anti‐FLAG magnetic agarose beads. Input and immunoprecipitates were analyzed by immunoblotting using anti‐FLAG and anti‐GFP antibodies. Experiments were performed three times with similar results.

CPK4/5/6/11 are extensively reported protein kinases involved in immunity (Bredow & Monaghan, 2019; Ormancey et al., 2017; Yip Delormel & Boudsocq, 2019). To investigate whether CPKs mediate the CBP60b‐regulated immune pathway, we examined the expression levels of shared downstream genes of the CBP60 family members, *EDS1* and *SID2*, as well as CBP60b‐specifically regulated genes *CBP60g* and *SARD1* (Li et al., 2021) in the *cpk4;5;6;11* mutant upon pathogen inoculation and observed downregulation of these genes in *cpk4;5;6;11* mutant (Figure S1), suggesting that CPKs may mediate CBP60b function. To test this hypothesis, we examined their potential interaction. Using bimolecular fluorescence complementation (BiFC) assays, we observed clear fluorescent signals in the nucleus for CPK4/5/6/11 with CBP60b (Figure 1A), whereas CPK28—another immune‐related CPK (Bao et al., 2025)—showed no detectable interaction signal with CBP60b in the nucleus, indicating the specific interaction between CBP60b and CPK4/5/6/11. Subsequently, *in vitro* pull‐down assays showed that MBP‐CBP60b could pull down His‐CPK4/5/6/11 (Figure 1B) but not His‐CPK28 (Figure S2). Finally, co‐immunoprecipitation (Co‐IP) experiments performed in leaf protoplasts further confirmed these results (Figure 1C; Figure S2), demonstrating that CBP60b and CPK4/5/6/11 are genuine interaction partners.

### CPK4/5/6/11 phosphorylate CBP60b

**Figure 2:**
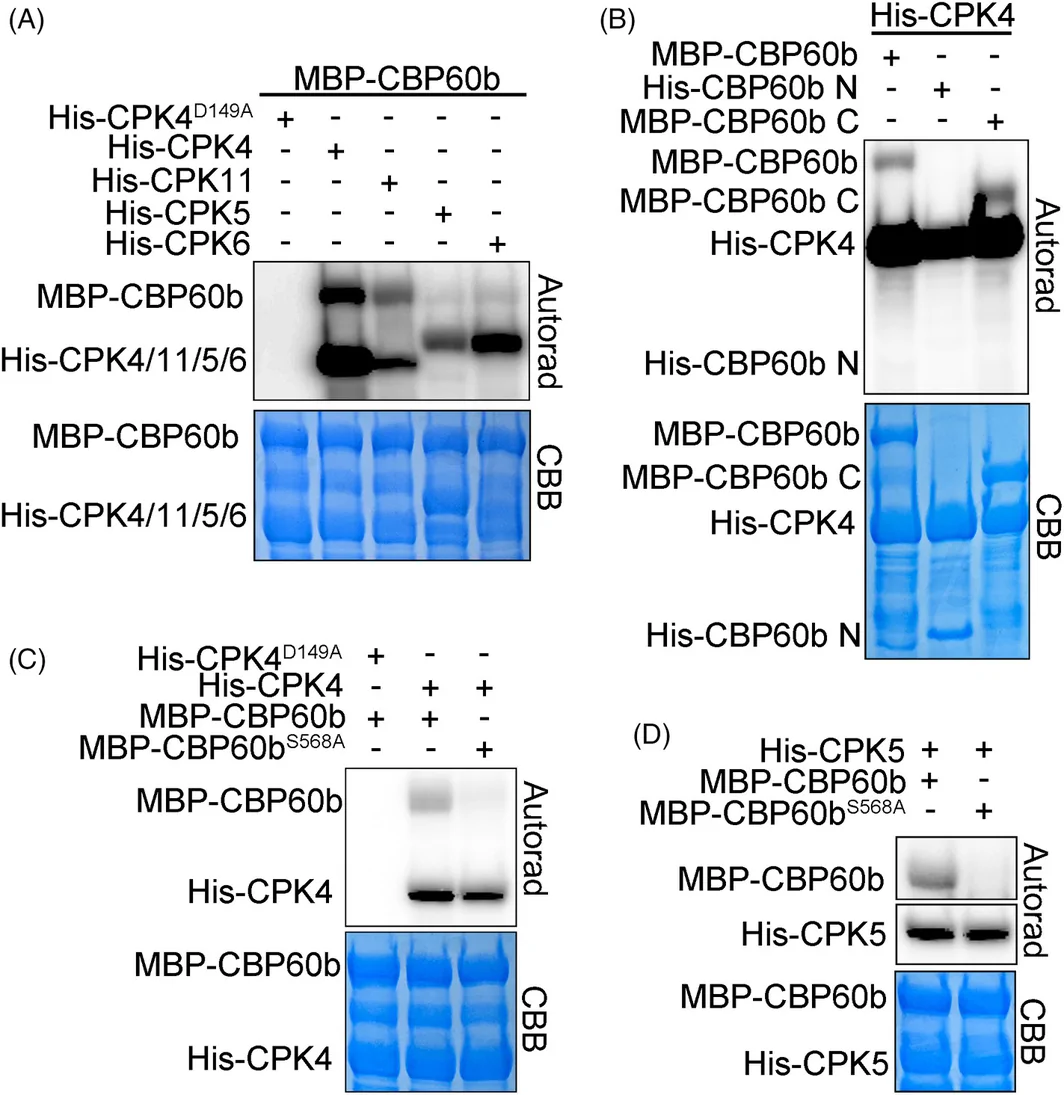
CPK4/5/6/11 phosphorylate CBP60b at ser568. (A) *In vitro* phosphorylation assays showing that CPK4/11/5/6 phosphorylate CBP60b. (B) Phosphorylation assays showing that CPK4 phosphorylates CBP60b C‐terminal. (C, D) CPK4 (C) and CPK5 (D) phosphorylate CBP60b at Ser568. Experiments were repeated two to three times with similar results. CBB, Coomassie Brilliant Blue staining of His‐tagged proteins. Experiments were performed three times with similar results.

Next, we investigated whether CBP60b serves as a phosphorylation substrate for CPKs. We found that CPK4/5/6/11 could phosphorylate CBP60b *in vitro* (Figure 2A). The CBP60b protein was divided into an N‐terminal fragment (1–221aa, containing the DNA‐binding motif) and a C‐terminal fragment (222–640aa, containing the transcriptional regulatory motif and the CaM‐binding domain). Phosphorylation assays revealed that the phosphorylation site resides in the C‐terminal region (Figure 2B). Previous studies reported that CPKs phosphorylate CBP60g at serine residues 450 and 495; however, these sites are not conserved in other CBP60 family members (Li et al., 2024). Based on mass spectrometry analysis, Ser568 was identified as a potential phosphorylation target of CPKs on CBP60b (Supporting Information Material S2). We then performed site‐directed mutagenesis and observed that phosphorylation signals of CBP60b^S568A^ by CPK4/5 were nearly abolished (Figure 2C,D), confirming that CPKs phosphorylate CBP60b at Ser568.

We encountered technical challenges when attempting to detect the phosphorylation status of CBP60b *in vivo*. Although previous studies have reported clear separation of phosphorylated and non‐phosphorylated CBP60g bands on Phos‐tag gels (Sun et al., 2022), our experimental system failed to produce distinctly resolved dual bands for CBP60b under similar conditions. We introduced the CBP60b‐GFP complementation construct (Li et al., 2021) into the *cpk4;5;6;11* mutant by hybridization, tentatively assigning the slower migrating diffuse band as phosphorylated CBP60b. Through multiple independent experiments, we consistently observed—in at least two replicates—that pathogen *Pseudomonas syringae pv. tomato* DC3000 (*Pto* DC3000)‐induced phosphorylation of CBP60b was significantly attenuated in the *cpk4;5;6;11* mutant (Figure S3). These findings suggest that CPKs are involved in regulating the phosphorylation of CBP60b in planta.

### Ser568 is essential for the transcriptional activity of CBP60b

**Figure 3:**
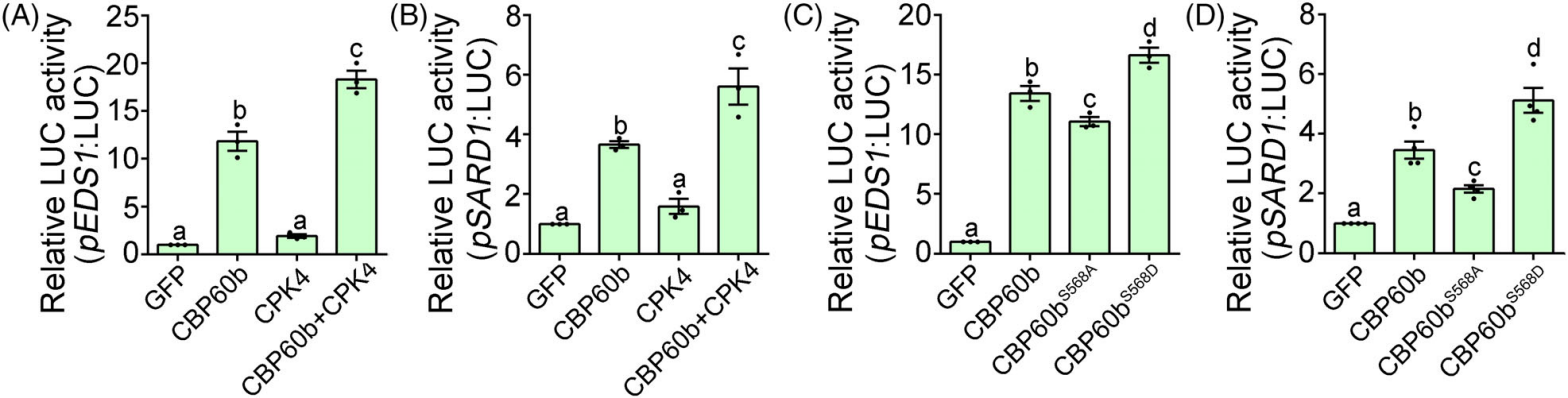
CPKs enhance the transcriptional activity of CBP60b through phosphorylation. (A, B) Quantitative luminescence showing the LUC activity of *proEDS1:LUC* (A) and *proSARD1:LUC* (B). The *promoter:LUC* reporter constructs were co‐transfected with *35S:GFP* (GFP), *35S:CBP60b‐GFP* (CBP60b), *UBQ10:CPK4‐GFP* (CPK4), or *35S:CBP60b‐GFP* along with *UBQ10:CPK4‐GFP* (CBP60b + CPK4). (C, D) Quantitative luminescence showing the LUC activity of *proEDS1:LUC* (C) and *proSARD1:LUC* (D) co‐transfected with *35S:GFP* (GFP), *35S:CBP60b‐GFP* (CBP60b), *35S:CBP60b* ^*S568A*^ *‐GFP* (CBP60b^S568A^), or *35S:CBP60b* ^*S568D*^ *‐GFP* (CBP60b^S568D^). LUC reporter activity was determined 16 h post‐transfection. Values are means ± SE (*n* = 3). Different letters indicate significantly different groups (1‐way ANOVA and Tukey's multiple comparison test; *P* < 0.05).

Ser568 resides within the transcriptional regulatory motif of CBP60b (Li et al., 2021), raising a possibility of its involvement in mediating CBP60b's transcriptional activity. To test this hypothesis, we performed LUC reporter assays. We found that co‐expression of CPK4 with the *promoter:LUC* reporter and CBP60b constructs enhanced CBP60b‐induced LUC activity driven by *EDS1* or *SARD1* promoters (Figure 3A,B). Furthermore, the phosphodeficient‐variant CBP60b^S568A^ decreased the induced LUC activity, whereas the phosphomimetic variant CBP60b^S568D^ increased it (Figure 3C,D). These results indicate that Ser568 is critical for the transcriptional activity of CBP60b.

To investigate whether CPKs influence the subcellular localization and protein accumulation of CBP60b, we observed the fluorescence of CBP60b‐GFP in the *cpk4;5;6;11* mutant. Compared to the wild type, no significant changes in the localization or fluorescence intensity of CBP60b were detected in the *cpk4;5;6;11* (Figure S4). These results suggest that CPKs specifically regulate the transcriptional activity of CBP60b without affecting its localization or protein stability.

### Ser568 is essential for CBP60b to respond to pathogens

**Figure 4:**
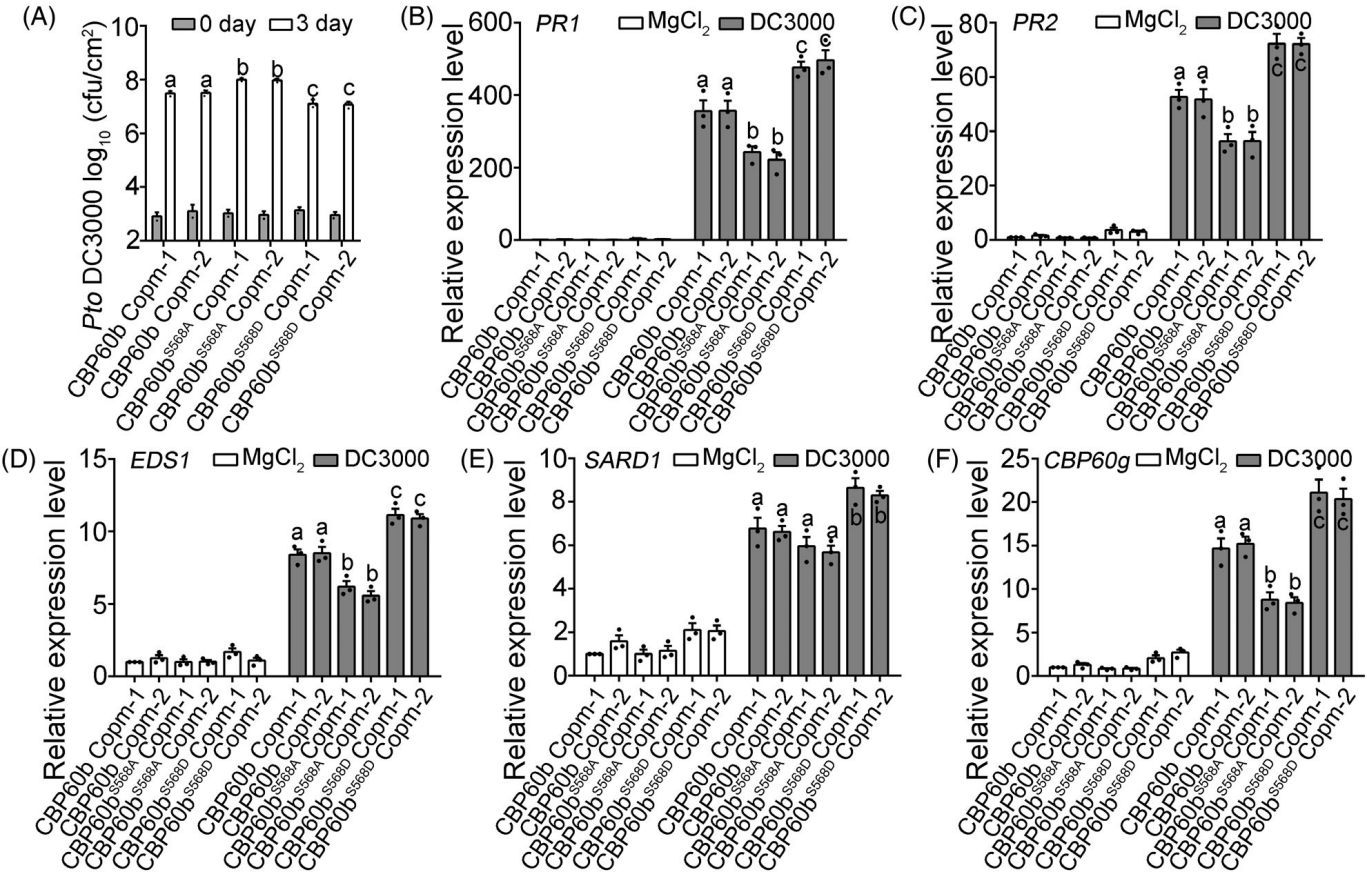
Ser568 phosphorylation of CBP60b is required for its response to pathogen. (A) Sensitivity of the indicated genotypes to *Pto* DC3000. Bacterial growth was determined 2 h post‐inoculation (0 days) or 3 days post‐inoculation (3 days). Values are means ± SD (*n* = 4). Experiment was repeated three times with similar results. (B–F) Relative transcript abundance of *PR1* (B), *PR2* (C), *EDS1* (D), *SARD1* (E) and *CBP60g* (F) upon mock treatment (MgCl_2_) or *Pto* DC3000 treatment in designated genotypes. RNAs were extracted from infiltrated leaves at 24 h post‐treatment. Results are means ± SE (*n* = 3). Different letters indicate significantly different groups (1‐way ANOVA and Tukey's multiple comparison test; *P* < 0.05).

To further investigate the functional impact of Ser568 on CBP60b, we generated *CBP60b* ^*S568A*^ *‐genomic‐GFP* and *CBP60b* ^*S568D*^ *‐genomic‐GFP* fusion constructs, introduced them into the *cbp60b* mutant, and selected transgenic lines with comparable exogenous CBP60b expression levels (Figure S5). These lines were found to fully complement the developmental defects of *cbp60b*, similar to the wild‐type CBP60b‐GFP control (Figure S6). Subsequently, we performed pathogen inoculation assays on these plants. Following inoculation, compared to the wild‐type CBP60b‐complemented plants, the CBP60b^S568D^ lines exhibited lower bacterial colony counts (Figure 4A), as well as higher expression levels of *EDS1*, *SARD1*, *CBP60g*, and immune marker genes such as *PR1* and *PR2* (Figure 4B–F). In contrast, the CBP60b^S568A^ lines showed the opposite effects (Figure 4), except that *SARD1* expression showed a slight but nonsignificant reduction (Figure 4E). Noting that, in the LUC reporter assay, the induction of the *SARD1* promoter by CBP60b^S568A^ was lower than that by CBP60b (Figure 3D), we speculate that other transcription factors (Kim et al., 2022) may be involved in pathogen‐induced *SARD1* expression *in vivo*, thereby compensating for the effect of CBP60b^S568A^ on *SARD1* expression. Taken together, these results demonstrate that phosphorylation of CBP60b at Ser568 is essential for its function in responding to hemibiotrophic bacterial pathogens.

### CBP60b regulates the gene expression of *CPK4/5/6/11*

**Figure 5:**
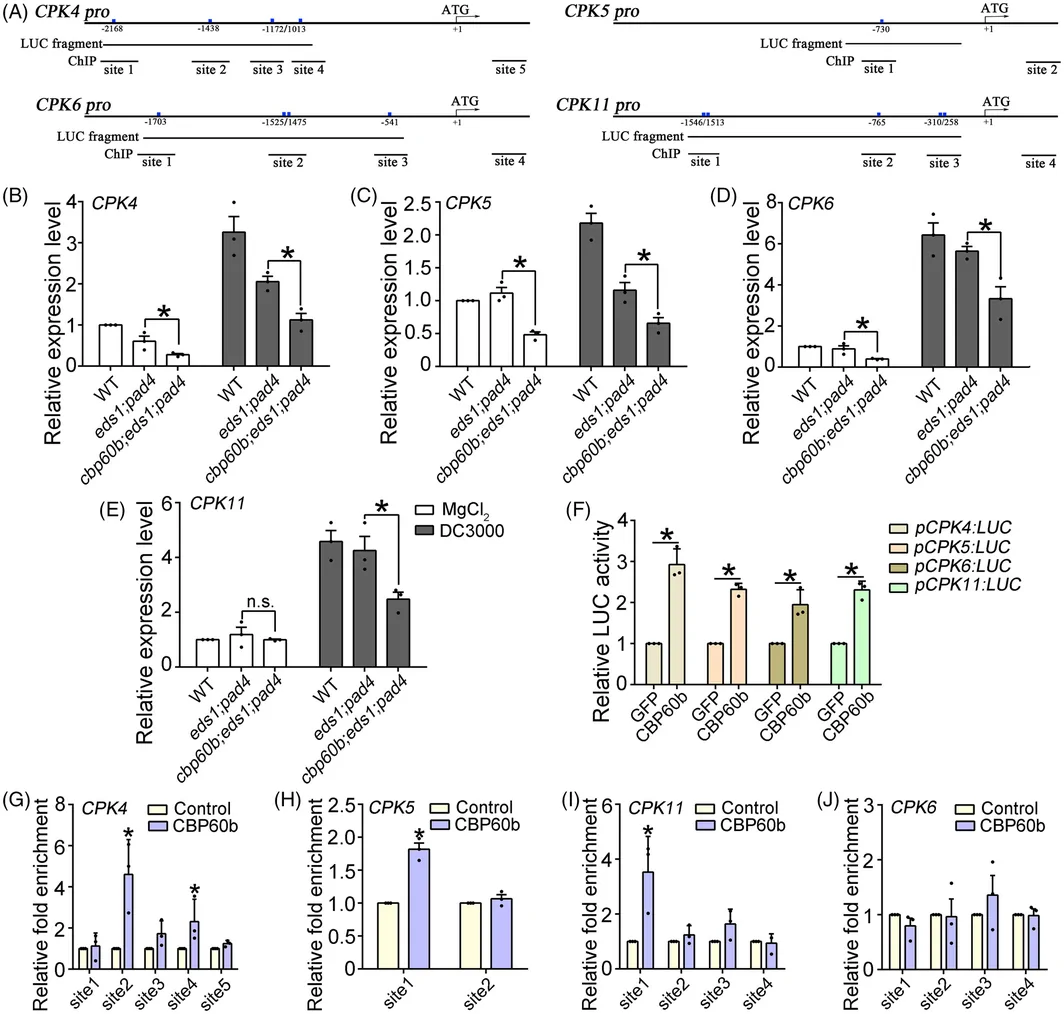
CBP60b regulates the expression of *CPK4/5/6/11*. (A) Schematic illustration of the promoter region of *CPK4/5/6/11*. +1 indicates the start of their responsive coding sequences. Blue boxes indicate the sites of G(A/T)AATT(T/G). LUC fragments and sites for ChIP PCRs are as indicated. (B–E) Relative transcript abundance of *CPK4* (B), *CPK5* (C), *CPK6* (D), and *CPK11* (E) upon mock treatment (MgCl_2_) or *Pto* DC3000 treatment in designated genotypes. RNAs were extracted from infiltrated leaves at 24 h post‐treatment. Results are means ± SE (*n* = 3). (F) Quantitative luminescence showing the LUC activity of *proCPK4/5/6/11:LUC* co‐transfected with *35S:GFP* (GFP) or *35S:CBP60b‐GFP* (CBP60b). Values are means ± SE (*n* = 3). (G–J) ChIP‐PCRs of CBP60b on *CPK4* (G), *CPK5* (H), *CPK11* (I) and *CPK6* (J). ChIP signal was quantified as the percentage of total input DNA by qPCR and normalized to corresponding fragment in WT (set as 1). Plants used are wild type (control) and CBP60b‐GFP (CBP60b) transformant plants. For *Pto* DC3000 treatment, plants at five WAG were infiltrated with *Pto* DC3000 (OD600 = 0.0001) 24 h before collecting and cross‐linking with 1% formaldehyde. Results are means ± SE (*n* = 3). Asterisks in (B–J) indicate significant difference (*t*‐test; *P* < 0.05).

The roles of CPK4/5/6/11 in immunity have been extensively documented (Bredow & Monaghan, 2019; Yip Delormel & Boudsocq, 2019); however, whether their expression is transcriptionally induced by the pathogen remains unclear. We found that *Pto* DC3000 inoculation significantly induced the expression of *CPK4/5/6/11* (Figure 5B–E). Analysis of the *CPK4/5/6/11* promoters revealed the presence of the G(A/T)AATT(T/G) motif recognized by CBP60 transcription factors (Sun et al., 2015) (Figure 5A). To determine whether CBP60b regulates *CPKs* expression, we performed qRT‐PCR and observed reduced expression levels of *CPK4/5/6/11* in the *cbp60b* mutant upon *Pto* DC3000 infection (Figure 5B–E). Furthermore, LUC reporter assays demonstrated that CBP60b induced LUC activity of *proCPK4/5/6/11* (Figure 5F). Chromatin immunoprecipitation (ChIP) assays confirmed that CBP60b directly binds to the promoters of *CPK4*, *CPK5*, and *CPK11 in vivo* (Figure 5G–I). Although CBP60b enrichment on the *CPK6* promoter was higher than the control, the difference was not statistically significant (Figure 5J). Given that CBP60 transcription factors recognize conserved binding motifs (Li et al., 2021; Sun et al., 2015) and that CBP60b regulates the expression of *CBP60g* and *SARD1* (Li et al., 2021), we hypothesized that CBP60b may also modulate *CPK6* expression indirectly through CBP60g and SARD1. Indeed, we found that CBP60g and SARD1 induced LUC activity of *proCPK6* (Figure S7). Collectively, these results provide substantial evidence that *CPK4*, *CPK5*, and *CPK11* are direct transcriptional targets of CBP60b.

## DISCUSSION

**Figure 6:**
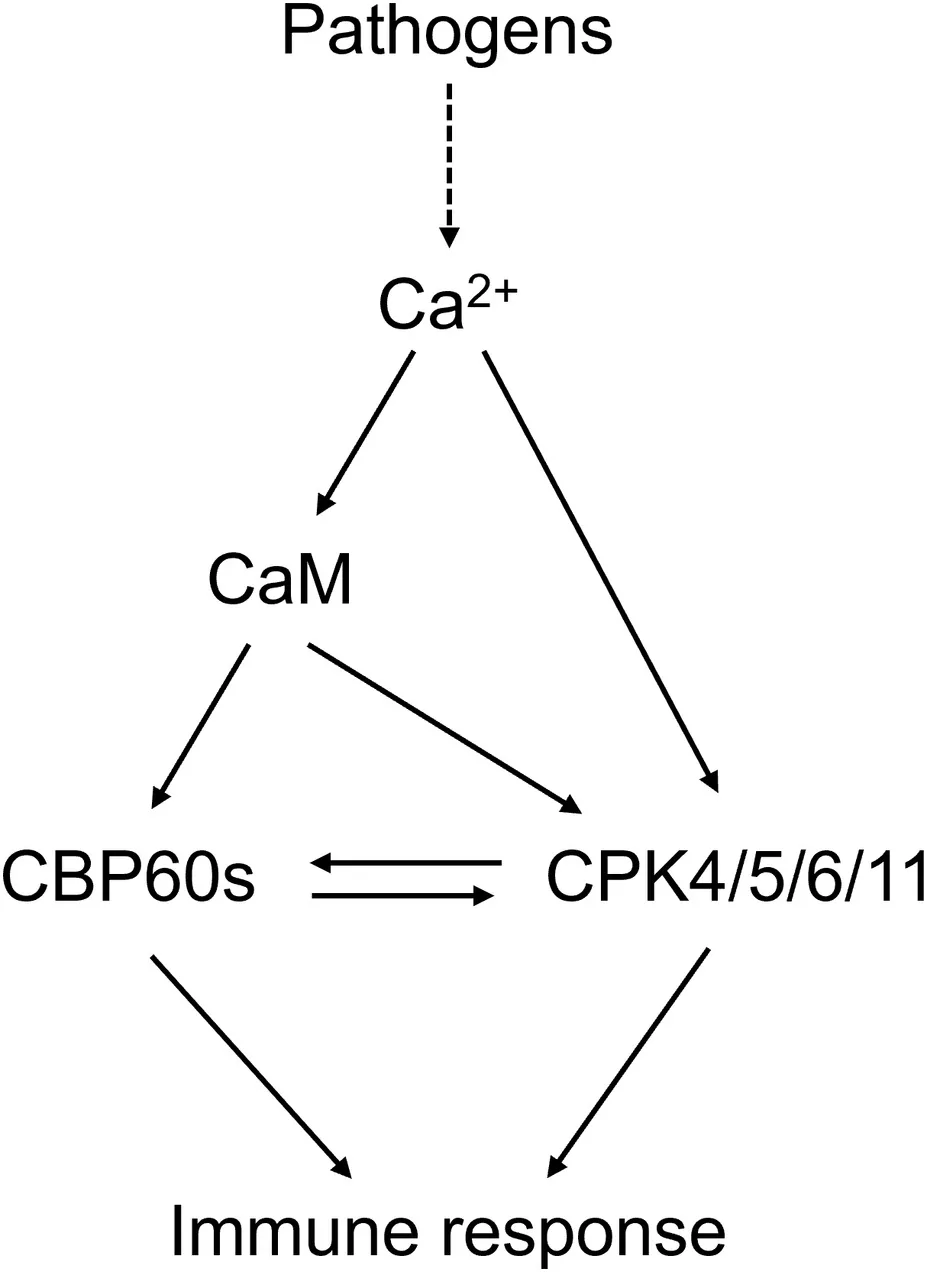
Working model: CBP60s‐CPK4/5/6/11‐positive feedback loop integrates Ca^2+^ signaling to respond to pathogen. CPK4/5/6/11 promote the activity of CBP60g and CBP60b through post‐translational phosphorylation, while CBP60s mediate pathogen‐induced *CPK4/5/6/11* expression at the transcriptional level. CBP60b and CBP60g are regulated by CaM, whereas CPK4/5/6/11 require Ca^2+^ and/or CaM to relieve autoinhibition and subsequently phosphorylate CBP60g and CBP60b. Thus, downstream of pathogen‐induced Ca^2+^ signaling, a CBP60s‐CPKs transcriptional‐phosphorylation positive feedback loop collectively mediates pathogen resistance.

In this study, we report that CBP60b and CPK4/5/6/11 form a positive feedback regulatory loop that mediates plant immune responses. Although the downstream genes and genetic pathways regulated by CBP60b have been previously characterized (Huang et al., 2021; Li et al., 2021; Li et al., 2024), post‐translational modification of CBP60b has not been reported. We provide the first demonstration that CBP60b undergoes phosphorylation through physical interaction with CPKs (Figures 1 and 2), which modulates its transcriptional activity without affecting its localization or protein turnover (Figures 3 and 4; Figure S4). Moreover, the pathogen‐induced transcriptional upregulation of *CPKs* during immunity is dependent on CBP60b, with *CPK4*, *CPK5*, and *CPK11* identified as direct transcriptional targets of CBP60b (Figure 5). Together, these findings establish a positive feedback loop that enhances plant defense against pathogens (Figure 6).

All CBP60 family members, with the exception of CBP60a, recognize and bind the promoter motif G(A/T)AATT(T/G) to regulate gene expression (Li et al., 2021; Li et al., 2024; Sun et al., 2015), conferring a degree of functional redundancy in their induction of gene expression. *CPK4* has been reported as a direct transcriptional target of CBP60g and SARD1 (Sun et al., 2015), and here, we demonstrate that *CPK4*, *CPK5*, and *CPK11* are direct targets of CBP60b *in vivo* (Figure 5). Moreover, the expression of *CPK6* was also found to be inducible by CBP60g and SARD1 (Figure S7). These findings indicate that multiple CBP60s coordinately induce the transcriptional expression of *CPKs* in response to pathogens, and that CBP60b regulates *CPKs* expression either by directly binding to *CPKs* promoter or indirectly through CBP60g and SARD1.

Concurrently, multiple CPKs physically interact with various CBP60s to mediate phosphorylation. It has been previously reported that CPK4/5/6/11 phosphorylate CBP60g at Ser450 and Ser495, thereby regulating its activity and resistance to the pathogen V. dahliae (Sun et al., 2022). In the present study, we demonstrate that CPK4/5/6/11 enhance the transcriptional activity of CBP60b through phosphorylation at Ser568 (Figure 3). These phosphorylation sites exhibit lineage‐specific conservation: Ser450/495 of CBP60g are not conserved in other CBP60 family members, and Ser568 of CBP60b is restricted to the CBP60b‐f clade (Li et al., 2024). Whether CPKs physically interact with and phosphorylate CBP60a or SARD1 remains unclear. Nonetheless, this pattern—shared physical interactions but divergent phosphorylation sites—may reflect an evolutionary “resilience” mechanism within the immune signaling network. In this scenario, functionally redundant positive regulators of immunity are diversified at critical residues to reduce the risk of being simultaneously hijacked by single pathogen effector, thereby enhancing the plant's capacity to defend against a broad spectrum of pathogens (Katagiri, 2023; Zheng et al., 2021).

We previously reported that the CBP60b clade is conserved throughout the evolution of land plant lineage, whereas other clade members emerged only in seed plants (Li et al., 2024; Zheng et al., 2021). The Ser568 residue is absent in CBP60 proteins from mosses and lycophytes, but is present in CBP60b orthologs from higher plant lineage such as tomato and cucumber (Li et al., 2024), suggesting that, as plants evolved, higher plant lineage developed refined regulatory mechanisms to cope with pathogen challenges. This phosphorylation event may, therefore, represent a regulatory strategy conserved in other higher plant species as well.

CBP60b and CBP60g are regulated by Ca^2+^‐CaM (Li et al., 2024; Reddy et al., 2002; Wang et al., 2009), and CaM has been shown to relieve the autoinhibition of CPK5, thereby enabling phosphorylation and activation of CBP60g (Sun et al., 2022). These findings indicate that Ca^2+^ signals activated during immune responses function through two parallel mechanisms: on the one hand, Ca^2+^ directly binds CPKs or facilitates CaM‐mediated relief of CPK autoinhibition, allowing phosphorylation of downstream targets such as CBP60b and CBP60g; on the other hand, Ca^2+^‐CaM directly enhances the transcriptional activity of CBP60g and the CBP60b clade. Thus, CBP60s act as central hubs in Ca^2+^ signal transduction, integrating and decoding spatiotemporally dynamic Ca^2+^ signals during immunity to fine‐tune transcriptional reprogramming (Figure 6). These discoveries further underscore the pivotal role of CBP60s in plant immune responses and provide a paradigm for investigating CBP60s and CPKs as a Ca^2+^‐decoding module in other plant species.

Given that the phosphodeficient variant CBP60b^S568A^ did not completely abolish transcriptional activity (Figure 3), and that it restored the growth defects of the *cbp60b* (Figure S6) but failed to restore its pathogen response (Figure 4), we conclude that CBP60b^S568A^ retains partial functionality yet has lost the capacity to respond to pathogens. How Ser568 mediates the transcriptional activity of CBP60b in the nucleus remains to be further elucidated and may involve the interaction of CBP60b with subunits of the mediator complex (An & Mou, 2013; Raya‐González et al., 2025). Additionally, given the roles of CPKs in regulating other transcription factors (Yip Delormel & Boudsocq, 2019), it is plausible that additional CPKs or other protein kinases modulate the subcellular localization or protein turnover of CBP60b—an intriguing and worthwhile topic for future investigation.

## MATERIALS AND METHODS

### Plant materials and growth conditions

*Arabidopsis thaliana* Columbia‐0 was used as the wild type. Plant materials including *cbp60b‐1*, *eds1;pad4*, *cbp60b;eds1;pad4*, *cpk4‐1;cpk11‐2*, *cpk5‐1;cpk6‐3*, *cpk4;5;6;11*, and *CBP60b‐GFP COM‐1/−2* lines were described previously (Li et al., 2021; Sun et al., 2022). Surface‐sterilized seeds were plated on half‐strength Murashige and Skoog (1/2 MS) basal medium plates, kept at 4°C in darkness for 3 days, then transferred to a growth chamber at 22°C for germination, and later transplanted to soil. Growth was maintained in a growth chamber at 22°C, 150 mE/m^2^s, 70% relative humidity under long day (LD) condition (16 h light/8 h dark) or short day (SD) condition (12 h light/12 h dark). Stable Arabidopsis transformations were performed using the floral dipping method. Transgenic plants were selected on 1/2 MS medium supplemented with 25 μg/mL Hygromycin (Roche).

### Plasmid construction

Expression vectors used in protein interaction assays were as followed. Constructs were generated using the Gateway™ technology (Invitrogen) unless noted otherwise. The pENTR/D/TOPO vector (Invitrogen) was used to generate entry vectors. The entry vector CBP60b and expression vector 35S:U1‐70K‐mRFP were described previously (Li et al., 2021). Primers for generating entry vectors were as follows: W1240a/W1241a for *CPK4*, W1237/W1238 for *CPK11*, W1496/W1498 for *CPK5*, W1574/W1575 for *CPK6* and W1820/W1821 for *CPK28*. Entry vectors were fused to destination vectors to generate *35S:nEYFP‐CBP60b*, *35S:CPK4‐cEYFP, 35S:CPK11‐cEYFP*, *35S:CPK5‐cEYFP*, *35S:CPK6‐cEYFP*, *35S:CPK28‐cEYFP*, *UBQ10:CPK4‐GFP*, *UBQ10:CPK5‐GFP*, *UBQ10:CPK6‐GFP*, and *UBQ10:CPK11‐GFP*, by LR reactions. Expression vectors used in pull‐down assays and *Flag‐CBP60b* by using the pEASY‐Uni Seamless Cloning and Assembly Kit (Transgen Biotech). Firstly, coding sequences of genes were amplified with the primer pairs: W1623/W1624 for *CPK4*, W1661/W1678 for *CPK5*, W1707/W1708 for *CPK6*, W1714/W1715 for *CPK11*, W1822/W1823 for *MBP‐CBP60b*, W1822/LW37 for *MBP‐CBP60b‐C*, LW38/LW39 for *His‐CBP60b‐C*, and LW9/LW10 for *Flag‐CBP60b*. Then, the resultant PCR fragment of *CPKs* and *CBP60b‐C* were recombined with the destination vector *pET‐32a* pre‐digested with BamHI/XhoI to generate *His‐CPK4/5/6/11* and *His‐CBP60b‐C*; the PCR fragments of *MBP‐CBP60b*, *MBP‐CBP60b‐C*, or *Flag‐CBP60b* were recombined with the destination vector *pMAL‐C2X* pre‐digested with BamHI/SalI or with the destination vector *pHBT‐Flag* pre‐digested with BamHI/StuI. Primers for generating mutational vectors were as follows: W1486/W1487 for *CPK4* ^*D149A*^, LW11/LW12 for *CBP60b* ^*S568A*^, and LW60/LW61 for *CBP60b* ^*S568D*^.

Expression vectors for transgenic plants and dual LUC reporter assays in Arabidopsis protoplasts were as follows. The expression vectors *35S:CBP60b‐GFP*, and *CBP60b genomic‐GFP* were described previously (Li et al., 2021). Promoter fragments used in LUC reporter assays were generated by primer pairs: LW1/LW2 for *CPK4*, LW3/LW4 for *CPK11*, LW5/LW6 for *CPK5*, LW7/LW8 for *CPK6*. PCR fragments of these promoters were inserted into pGreen II‐0800 vector (Li et al., 2021) predigested with KpnI/SalI using pEASYUni Seamless Cloning and Assembly Kit to generate LUC reporter constructs.

All PCR amplifications used PhusionTM hot start high‐fidelity DNA polymerase at the annealing temperature and extension times recommended by the manufacturer. The Bioneer PCR purification kit and Bioneer Spin miniprep kit were used for PCR product recovery and plasmid DNA extraction, respectively. All constructs were sequenced and analyzed using Vector NTI. Primers are listed in Supporting Information Material S1.

### BiFC assays

The vectors *35S:CPK4‐cEYFP*, *35S:CPK11‐cEYFP*, *35S:CPK5‐cEYFP*, *35S:CPK6‐cEYFP*, *35S:CPK28‐cEYFP*, *35S:nEYFP‐CBP60b*, and *35S:U1‐70K‐mRFP* were transformed into *A. tumefaciens* strain GV3101 as described (Li et al., 2024). After overnight grown at 28°C, *A. tumefaciens* were harvested by centrifuging at 2500 ***g*** for 10 min and suspended in MES medium to OD600 of 2.0. Next, the two types of cultures containing the cEYFP‐ or nEYFP‐fused plasmids were equally mixed with *A. tumefaciens* transformed with *35S:U1‐70K‐mRFP* and P19, kept at room temperature for 2–4 h in the dark before infiltrating them to the abaxial side of *N. benthamiana* leaves using 1‐mL disposable syringes. Plants were kept in a 16‐h light:8‐h dark cycle at 22°C for 48 h before confocal imaging. For every BiFC combination, at least 30 U1‐70K‐mRFP‐positive epidermal cells from three biological replicates were examined for eYFP signals.

### Pull‐down assays

The His‐ and MBP‐fused recombinant vectors were expressed in BL21 (DE3) strain and purified using Ni‐NTA agarose (Molecular Cloning Laboratories) or amylose resin (New England Biolabs), respectively, according to the manufacturer's instructions. Purified His, His‐CPKs, and MBP‐CBP60b protein were co‐incubated with 10 μL amylose magnetic beads (PuriMag Pro) in binding buffer (20 mM Tris–HCl, 0.2 M NaCl, 1 mM EDTA, 1 mM DTT, pH = 7.4) at 4°C for 2 h. After washing five times with binding buffer, the proteins were separated by SDS‐PAGE and transferred to a membrane. The membrane was incubated with primary antibody (Transgen Biotech, anti‐MBP and anti‐His, 1:5000) for 2 h at room temperature and then with mouse secondary antibody for 1 h at room temperature. Immunoreactive signals were visualized using a VILBER Fusion Pulse 6 system.

### Co‐immunoprecipitation (Co‐IP) assays

Four‐week‐old Arabidopsis plants were used for protoplast isolation as described (Li et al., 2024). pHBT‐Flag‐CBP60b was co‐transfected with UBQ10:GFP or UBQ10:CPK4/5/6/11‐GFP into Arabidopsis protoplasts. Total protein was extracted with extraction buffer containing 50 mM Tris–HCl (pH = 7.5), 150 mM NaCl, 1 mM EDTA, 1% Triton X‐100, and protease inhibitor cocktails. For anti‐Flag IP, total protein was incubated with 10 μL of anti‐Flag nanobody magarose beads (AlpalifeBio) at 4°C for 2 h. The bound beads were washed five times with wash buffer containing 50 mM Tris–HCl (pH = 7.5), 150 mM NaCl, 1 mM EDTA, collected, and boiled for 5 min with protein loading buffer. The proteins were separated by SDS‐PAGE and transferred to a membrane. The membrane was incubated with primary antibody (Transgen Biotech, anti‐Flag and anti‐GFP, 1:5000) for 2 h at room temperature and then with mouse secondary antibody for 1 h at room temperature. Immunoreactive signals were visualized using a VILBER Fusion Pulse 6 system.

### Phosphorylation assays

Phosphorylation assays were performed as described (Sun et al., 2022). *In vitro* phosphorylation reactions were performed in 20‐μL reaction buffer containing 20 mM Tris–HCl (pH = 7.2), 10 mM MgCl_2_, 5 mM CaCl2, and 1 mM DTT. His‐CPK4, His‐CPK4^D149A^, His‐CPK5, His‐CPK6, or His‐CPK11 was incubated with MBP‐CBP60b, respectively, in reaction buffer supplied with 0.1 mM ATP and 5 μCi of [γ‐^32^P] ATP at 30°C for 30 min. The reactions were stopped by adding 5 μL 5X SDS loading buffer and incubation at 100°C for 10 min. After SDS‐PAGE separation and Coomassie Brilliant Blue (CBB) staining, dried gels were tightly attached inside phosphor exposure cassette, followed by storage phosphor screen contact exposure at room temperature in dark. The exposed phosphor screen was scanned using a Typhoon 9410 Variable Mode Imager (Amersham Biosciences) in Phosphor Imaging mode. The scanning parameters were: pixel resolution 50 μm, maximum acquisition sensitivity, and the instrument’s automatic default PMT and laser settings for phosphor screens. The scan area was manually defined based on the gel dimensions. Scanned raw .gel files were exported by ImageQuant TL software.

### Phos‐tag SDS PAGE

The SDS‐PAGE gel was supplemented with 30 mM Phos‐tag and 60 mM MnCl_2_ for Phos‐tag SDS‐PAGE. Following electrophoresis, the gel was washed three times with 10 mM EDTA before protein transfer. The membrane carrying the transferred protein was immunoblotted with an anti‐GFP antibody (1:5000). Band intensities (integrated density) for both phosphorylated and non‐phosphorylated protein bands were quantified with ImageJ. The relative phosphorylation level was then calculated as the ratio of the non‐phosphorylated band to the phosphorylated band.

### Arabidopsis mesophyll protoplast isolation and transfection

Leaves of four WAG Arabidopsis plants grown under 16 h light/8 h dark were harvested and protoplasts were isolated following the tape‐Arabidopsis sandwich method (Wu et al., 2009). Protoplasts were co‐transformed with various combinations of constructs using the PEG/calcium‐mediated method (Yoo et al., 2007). An equal volume of freshly prepared PEG4000 solution containing 40% (w/v) PEG, 0.1 M CaCl_2_, and 0.2 M mannitol was added, completely mixed, and incubated at RT for 10 min. A 440 μL of modified W5 solution (154 mM NaCl, 125 mM CaCl_2_, 5 mM KCl, and 2 mM MES) was added and gently mixed to stop the transfection. Transfected protoplasts were collected by centrifugation at 100 ***g*** for 2 min and were resuspended in 0.5 mL of W5 solution. The final protoplasts were incubated in a 1% BSA precoated 12‐well plate at 22°C for 16 h in light and then used for CoIP or LUC reporter assays.

### Dual luciferase reporter assays in Arabidopsis protoplasts

Transfected protoplast suspension was transferred to a 1.5‐mL centrifugation tube and centrifuged at 100 ***g*** for 10 min. The supernatant was discarded and the pellets were re‐suspended in 50 μL of 1X lysis buffer provided in the double‐luciferase reporter assay kit (TransGen Biotech). Protoplasts were disrupted by vortex for 30 s followed by centrifugation at 10000 ***g*** for 2 min. A 5‐μL of the supernatant sample was loaded into a well of a 1.5‐mL centrifugation tube. Luciferase activity was tested with a double‐luciferase reporter assay kit (TransGen Biotech) according to the manufacturer's instructions, using the dual‐light chemiluminescent reporter gene assay system (Berthold). The ratio of LUC driven by the promoters to Renilla LUC (REN) driven by the 35S promoter was calculated to determine the transcriptional activities. All experiments were repeated in three to four biological replicates.

### Pathogen inoculation and bacterial growth assays

*Pto* DC3000 strains were cultured at room temperature (RT) in King's B medium (protease peptone, 10 mg/mL; glycerol, 15 mg/mL; K_2_HPO_4_, 1.5 mg/mL; MgSO_4_, 5 mM, pH 7.0) supplemented with 12.5 mg/mL rifampicin. Plants at five WAG under SD condition were used for inoculation. *Pto* DC3000 suspensions in 10 mM MgCl_2_ (OD_600_ = 0.0001) were infiltrated into the abaxial sides of mature leaves using a needleless 1‐mL syringe. To determine the bacterial titers (CFU/cm^2^), leaves were sampled at 2 hrs and 3 days by 0.5‐cm diameter puncher. Samples were grinded, serially diluted 10‐fold and plated on King's B medium plates. The number of colonies was counted after 2 days of culture at RT.

### RT‐PCRs and RT‐qPCRs

Total RNAs were extracted from fifth to sixth true leaves after five WAG plants under SD condition except where noted. All experiments were repeated in three to four biological replicates. Each biological replicate includes 8–10 leaves from five plants. Total RNAs were isolated using Ultrapure RNA Kit (Cwbiotech) according to the instruction of manufacturers. Reverse transcription was performed with ReverTra Ace qPCR RT Master Mix with gDNA Remover (Toyobo). RT‐qPCRs were performed with the ABI QuantStudio™ 6 Flex using SYBR Green real‐time PCR master mix (Toyobo). *GAPDH* was used as a quantitative control for RT‐qPCRs. The relative expression of genes was analyzed by the 2^−∆∆Ct^ method. All primers are listed in Supporting Information Material S1.

### ChIP

The EpiQuik Plant ChIP kit (Epigentek, Farmingdale, NY, USA; P‐2014) was used to perform the ChIP assays (Li et al., 2021). Approximately 1.0 g of leaf tissue was harvested from uninoculated or *Pto* DC3000 (OD600 = 0.0001)‐inoculated CBP60b‐GFP plants at 24 h after infiltration. Chromatin was immunoprecipitated with an anti‐GFP antibody (TransGen Biotech). The primers used for qPCRs are listed in Supporting Information Material S1. All experiments were repeated in three biological replicates.

### Fluorescence imaging

Fluorescence imaging of CBP60b‐GFP was performed with a Zeiss LSM880 (Zeiss) confocal laser scanning microscope (CLSM). For CLSM imaging of GFP, excitations/emissions are 488 nm/513 nm. Fluorescent images in BIFC experiments were captured using a Zeiss LSM 880 CLSM with 1024*1024 Pixels. For confocal laser scanning micrograph imaging of YFP and mRFP, excitations/emissions are 514 nm/520–550 nm and 561 nm/575–650 nm, respectively. Double fluorescent‐labeled materials were captured alternately using line‐switching with the multi‐track function.

### Western blot assays

Western blot analysis was performed as described (Li et al., 2021). Anti‐GFP antibody (1:2000, TransGen Biotech) was used to recognize CBP60b‐GFP while anti‐Actin (1:2000, TransGen Biotech) was used as the internal loading control. The antibody incubation conditions were as described above.

## Accession Numbers

Arabidopsis genes involved can be found in TAIR under the following accession numbers: AT4G09570 for *CPK4*, AT4G35310 for *CPK5*, AT2G17290 for *CPK6*, AT1G35670 for *CPK11*, AT5G66210 for *CPK28*, AT5G57580 for *CBP60b*, AT5G26920 for *CBP60g*, AT1G73805 for *SARD1*, AT3G48090 for *EDS1*, AT3G52430 for *PAD4*, AT1G74710 for *SID2*, AT2G14610 for *PR1*, AT3G57260 for *PR2*, and AT3G04120 for *GAPDH*.

## Author Contributions

LSL initiated the project; LSL and LM conducted all experiments with the technical assistance of YXC; LSL analyzed data and wrote the article; SL and YZ supervised this project; LSL and SL provided funding.

## CONFLICT OF INTEREST

The authors declare no conflict of interest.

## Supporting information


**Figure S1.** The expression of immune genes induced by pathogens is reduced in *cpk4;5;6;11*.
**Figure S2.** CBP60b interacts with CPK4 but not with CPK28.
**Figure S3.** Pathogen‐induced phosphorylation of CBP60b is reduced in *cpk4;5;6;11*.
**Figure S4.** CPKs do not affect the localization and protein accumulation of CBP60b.
**Figure S5.** The expression level of exogenous CBP60b is relatively consistent.
**Figure S6.** CBP60b^S568A^ and CBP60b^S568D^ rescue the developmental defects of *cbp60b*.
**Figure S7.** CBP60g and SARD1 induce *CPK6* expression.

**Data S1.** Oligos used in this study.

**Data S2.** MS/MS spectrum of the peptide containing phosphorylated S568 by LC‐MS/MS analysis.

**Data S3.** Uncropped blots in all figures.

**Data S4.** Source data in all figures.

## Data Availability

All data generated or analyzed during this study are included in this published article and its Supporting Information materials.
