# Supplementary material for: The CPKs‐CBP60b positive feedback loop regulates immunity in Arabidopsis
Source: Plant J. 2026 Sep 8;127(5):e71100. doi: 10.1111/tpj.71100 (PMC13553119; doi:10.1111/tpj.71100)

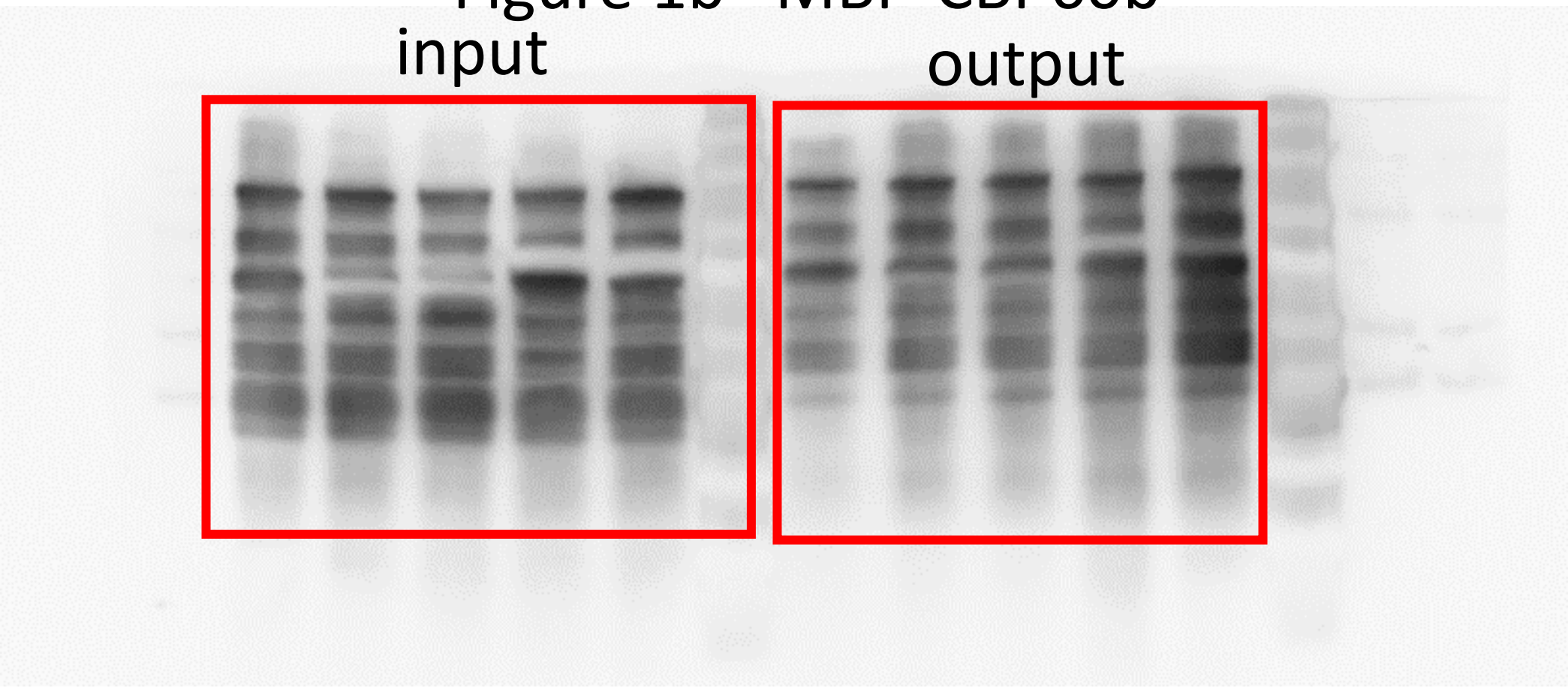
Figure 1b MBP-CBP60b
input
output

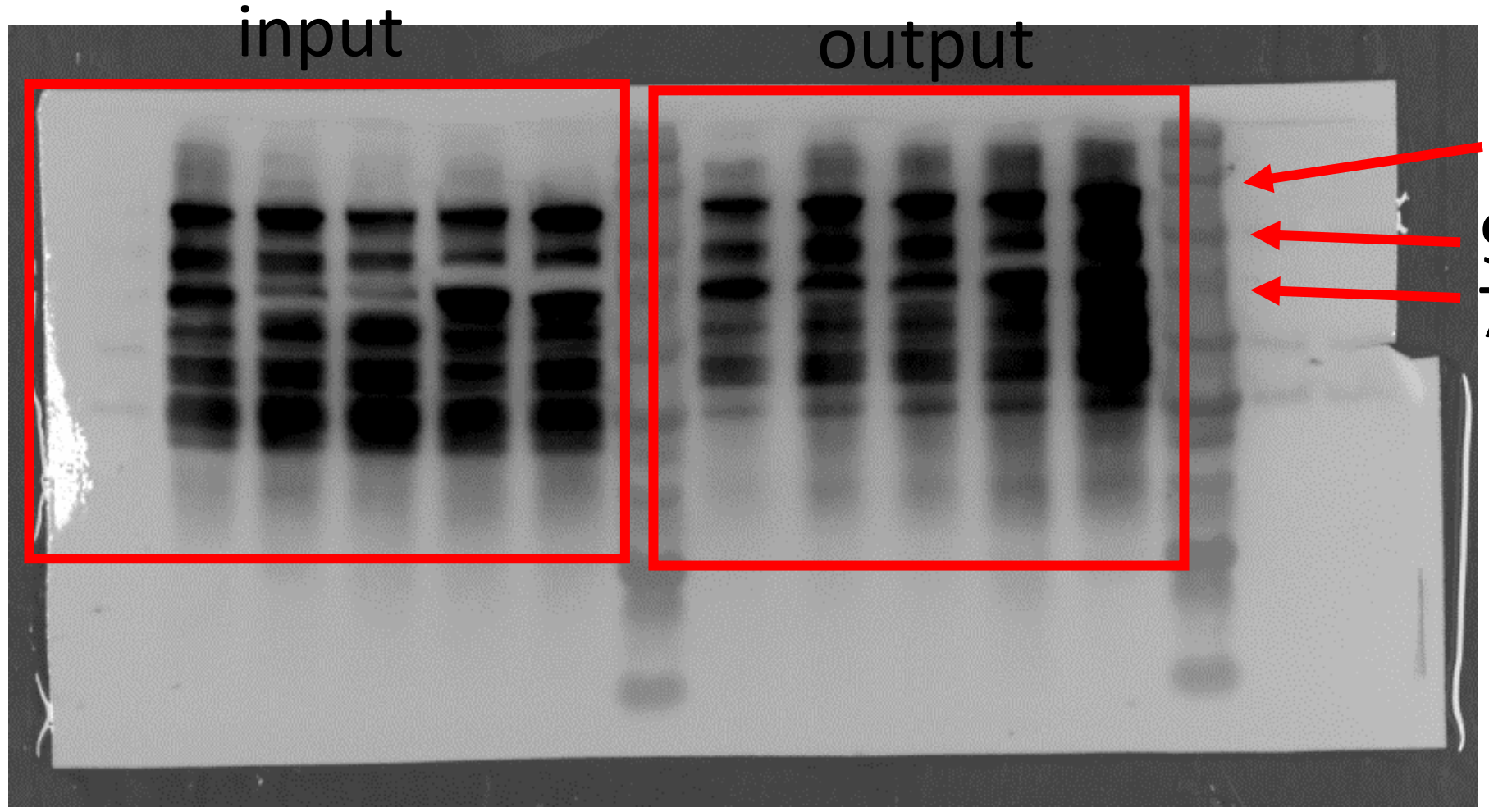
Figure 1b MBP-CBP60b
input
output
135kDa
98kDa
75kDa

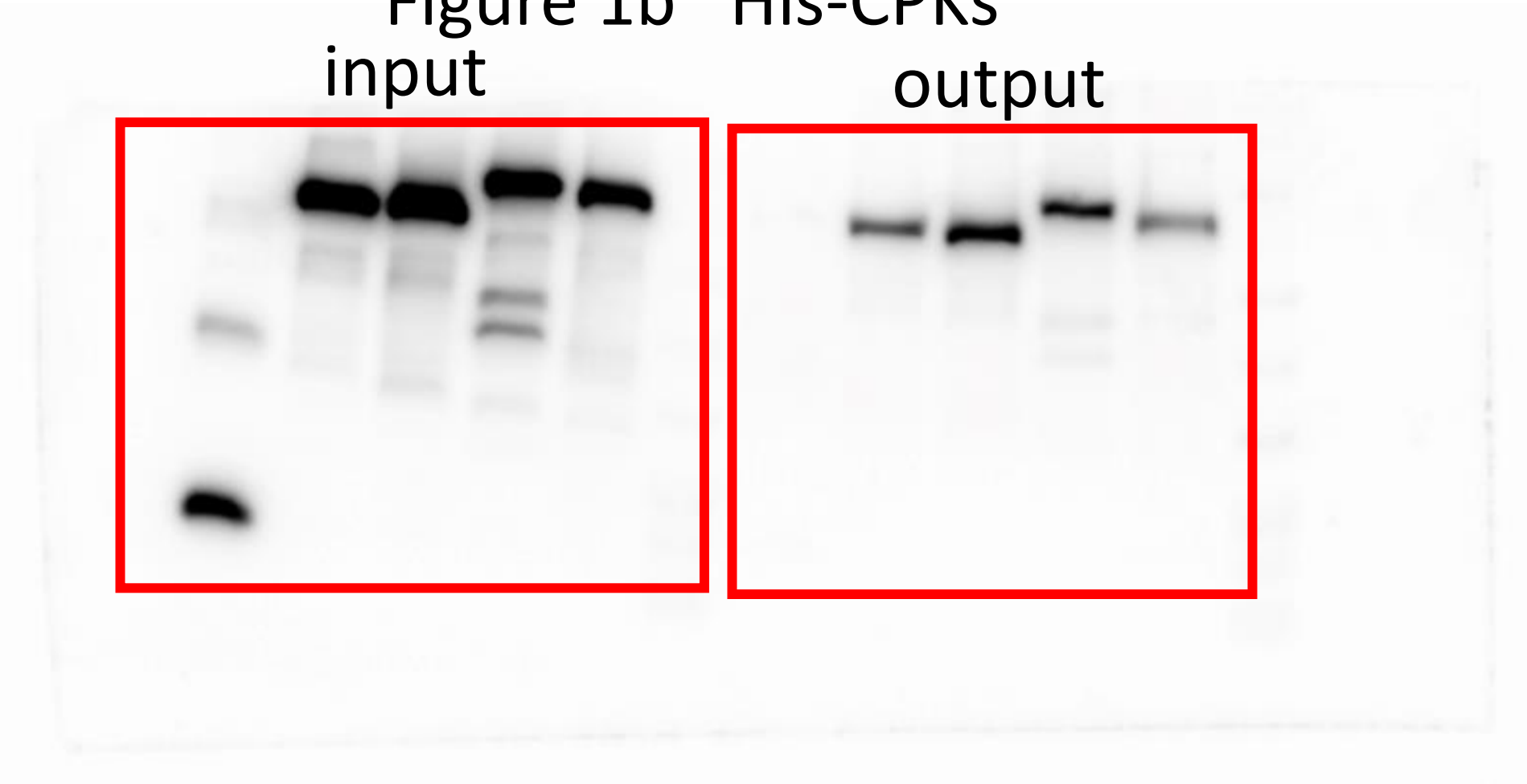
Figure 1b His-CPKs
input
output

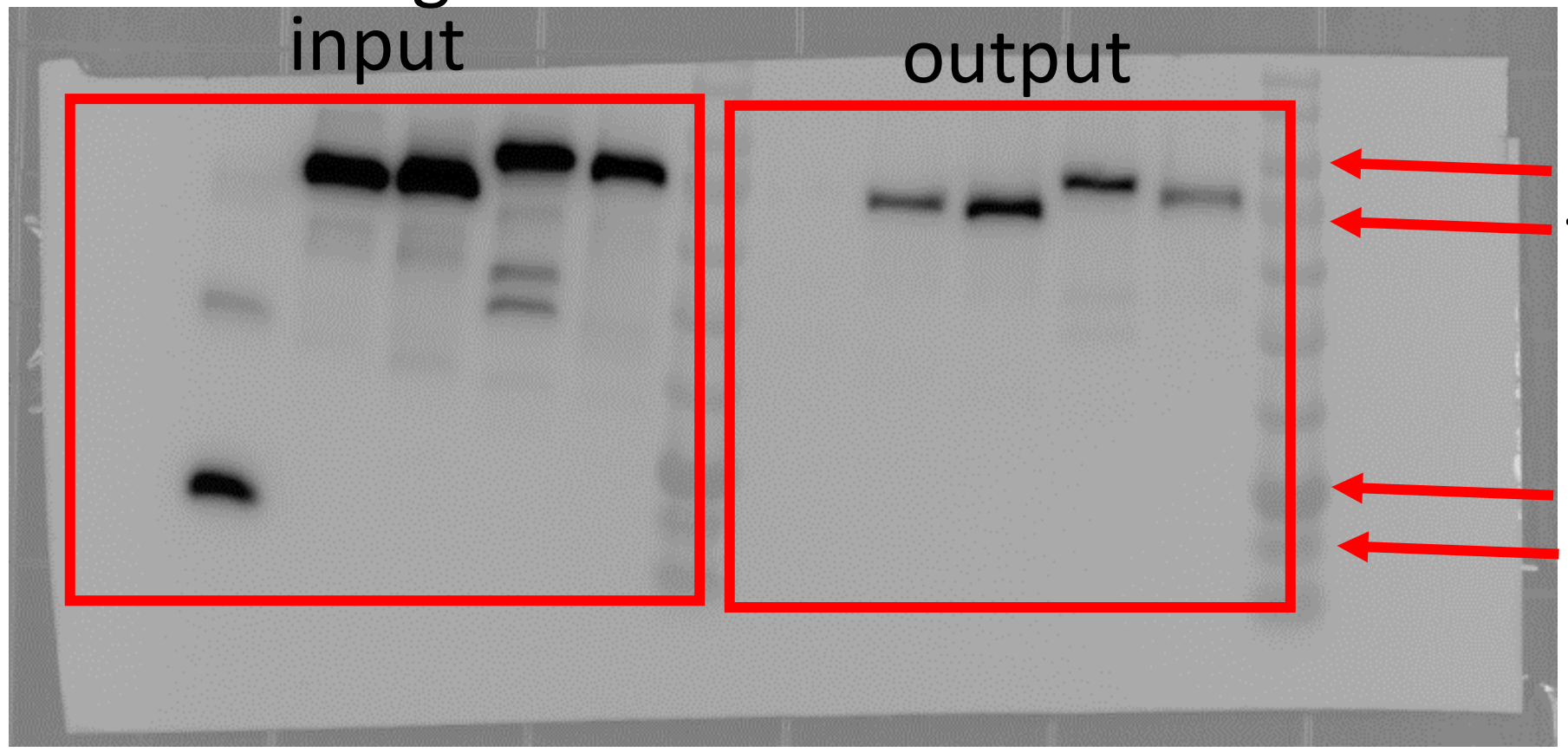
Figure 1b His-CPKs
input
output
98kDa
75kDa
25kDa
17kDa

Figure 1c Flag-CBP60b input

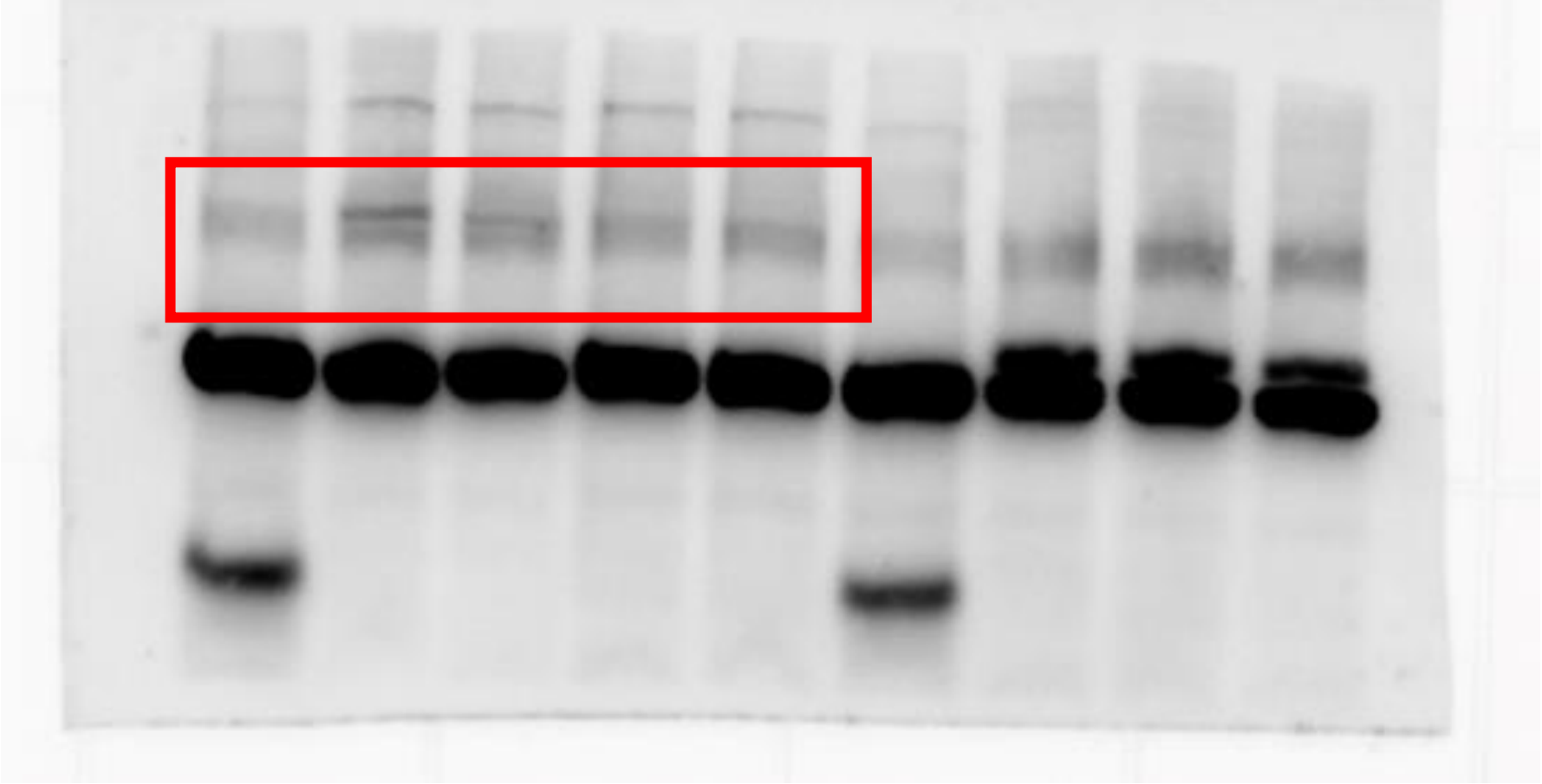

Figure 1c Flag-CBP60b input

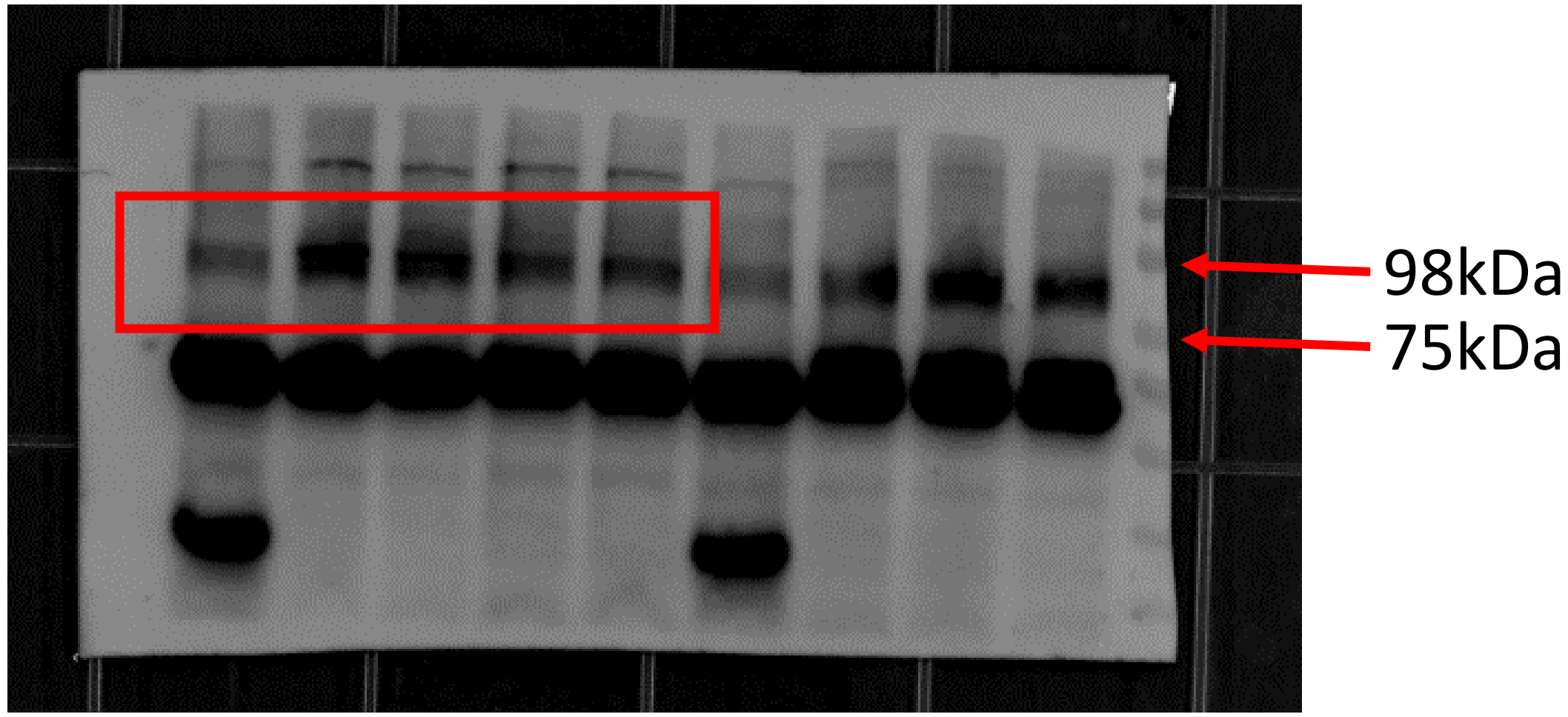

Figure 1c CPKs-GFP input

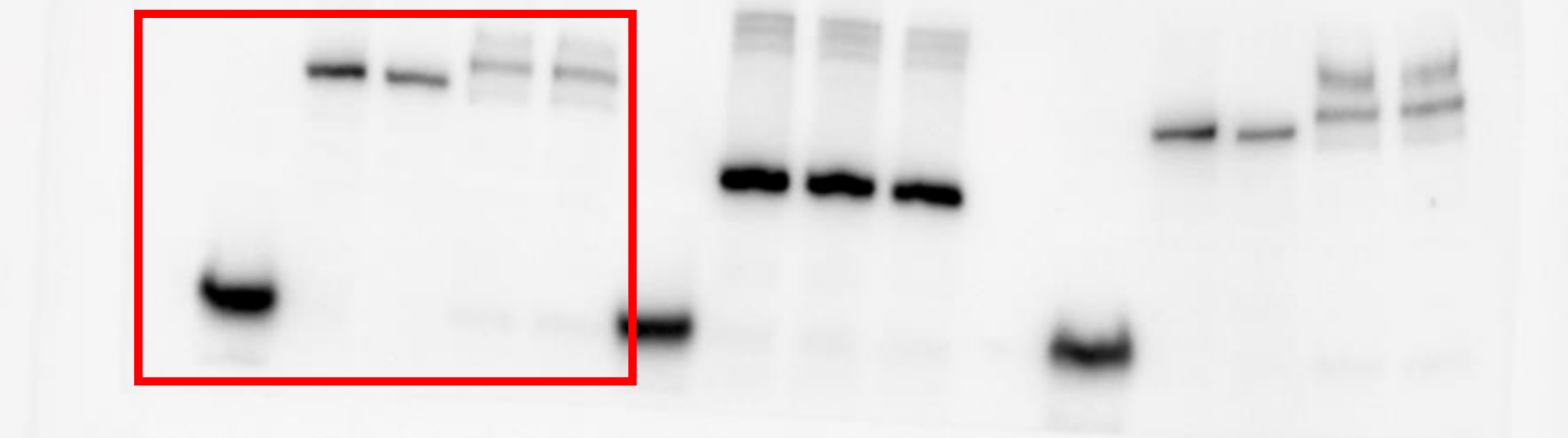

Figure 1c CPKs-GFP input

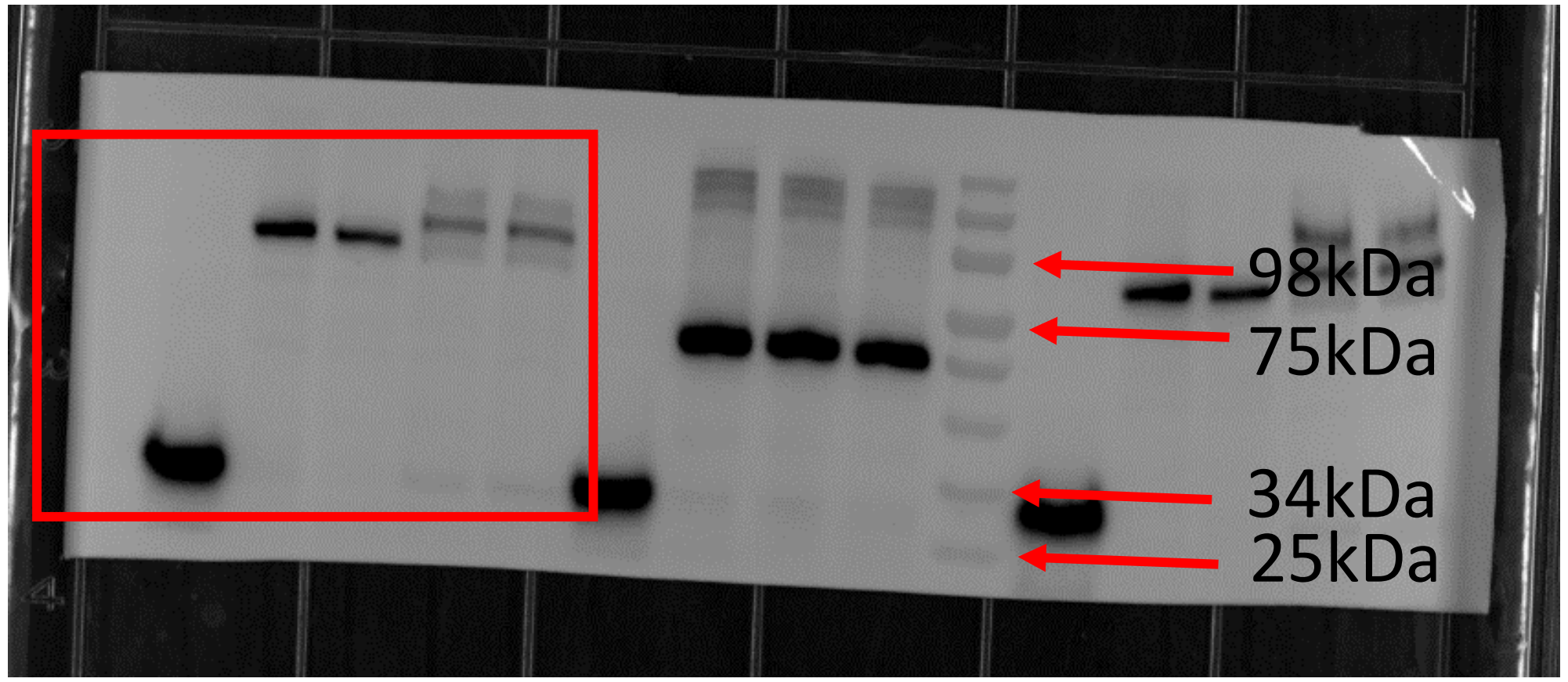

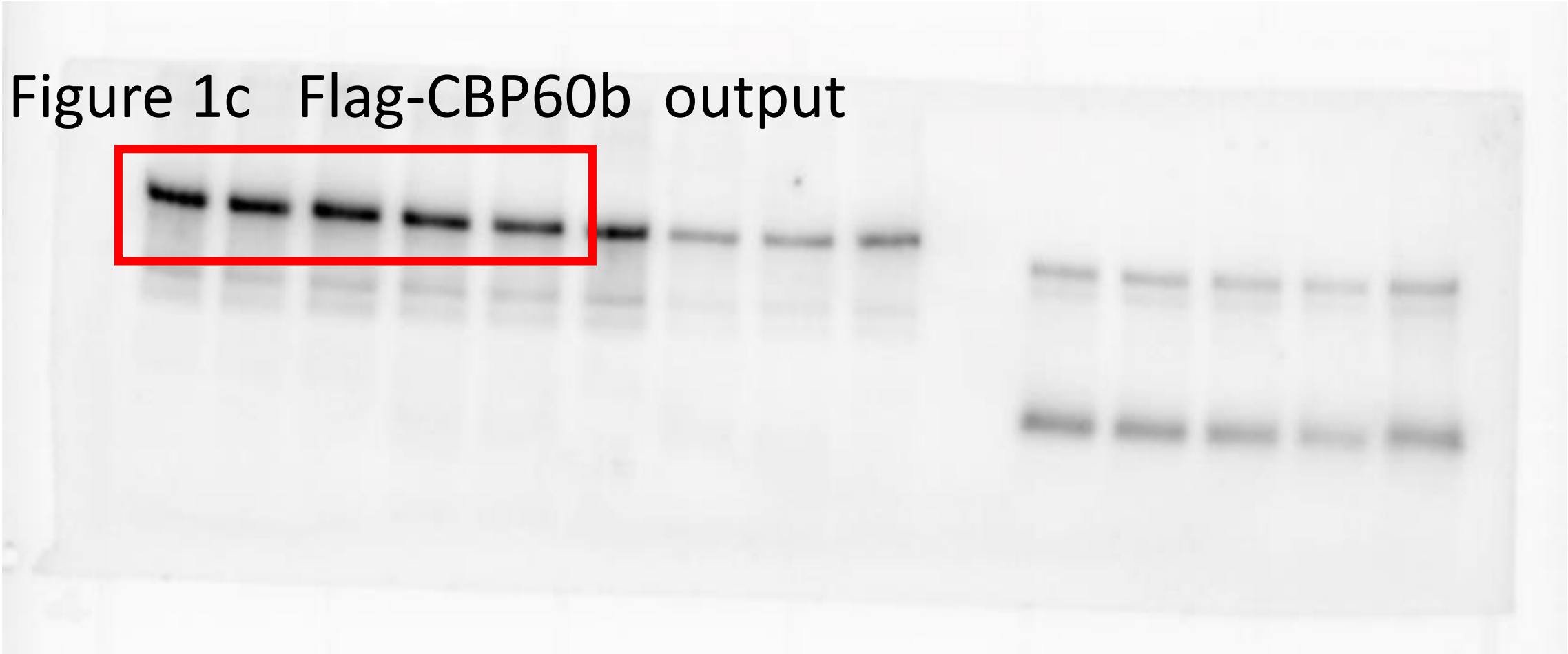
Figure 1c Flag-CBP60b output

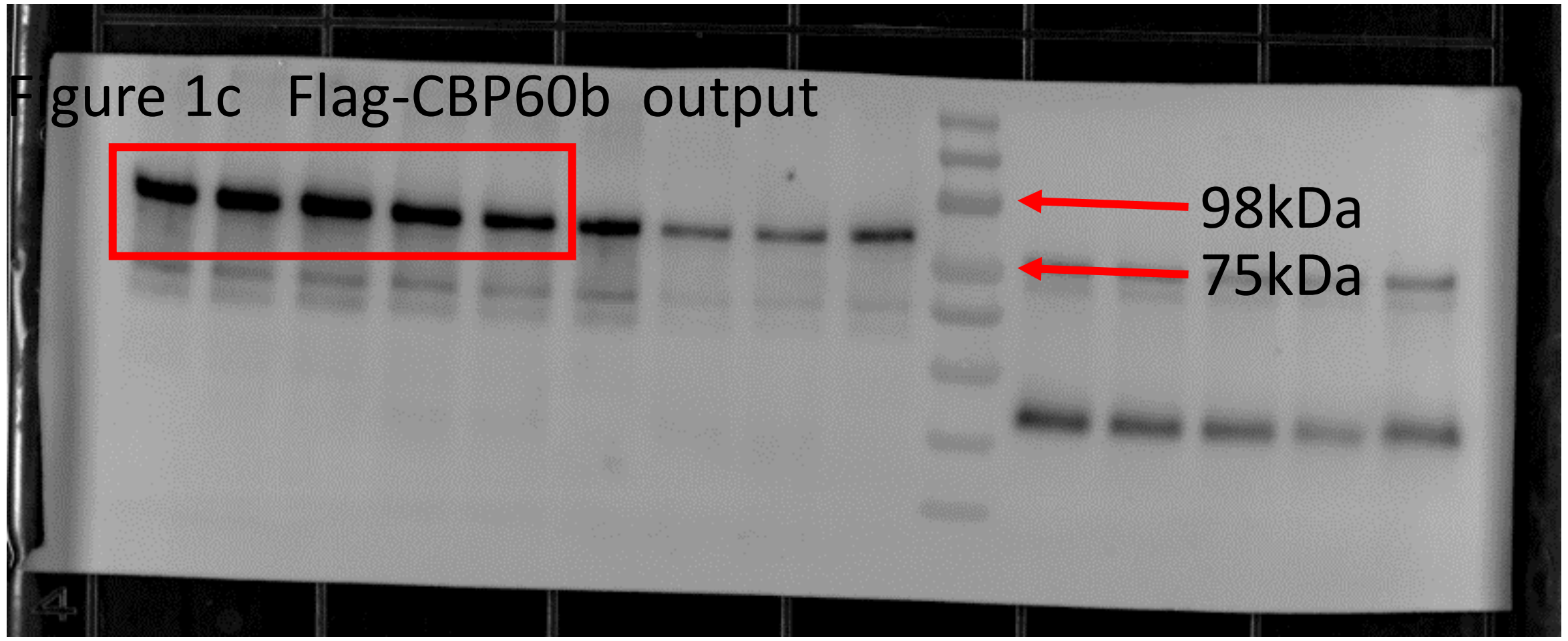
Figure 1c Flag-CBP60b output
98kDa
75kDa

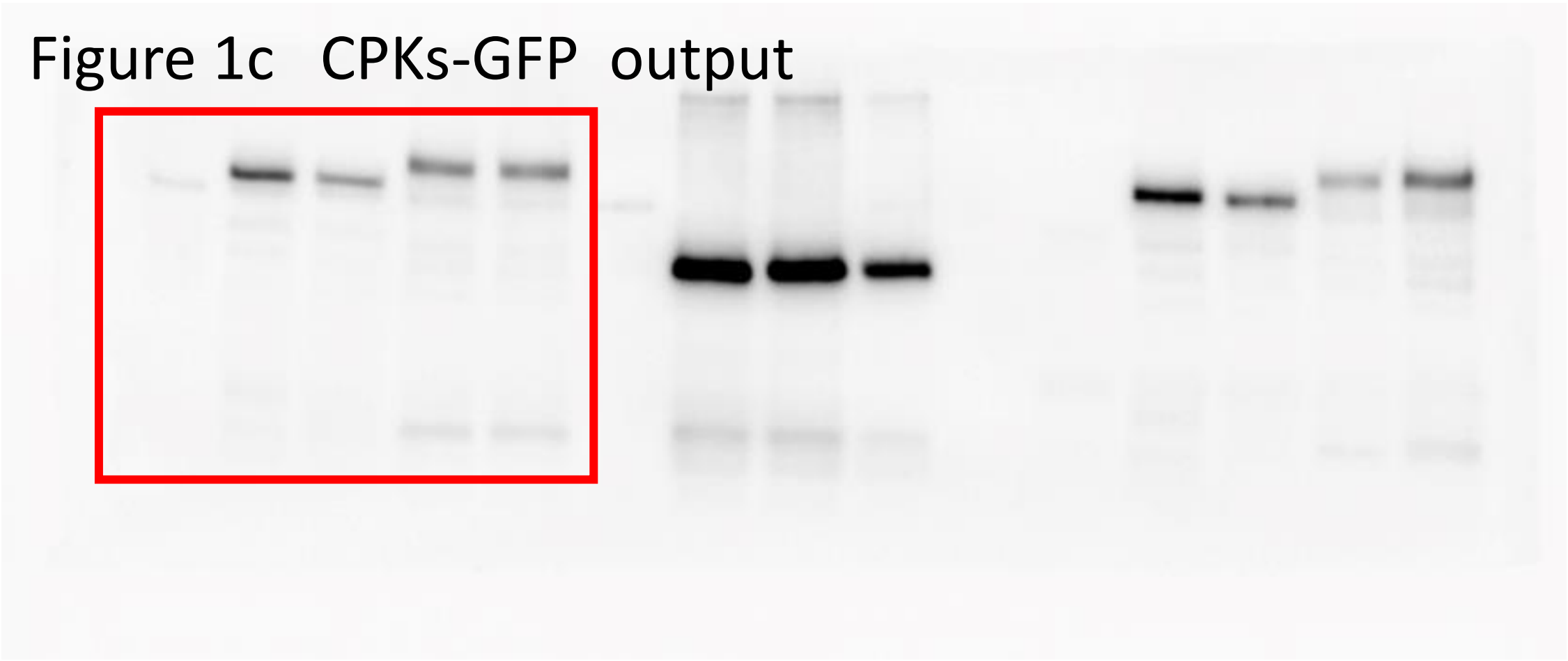
Figure 1c CPKs-GFP output

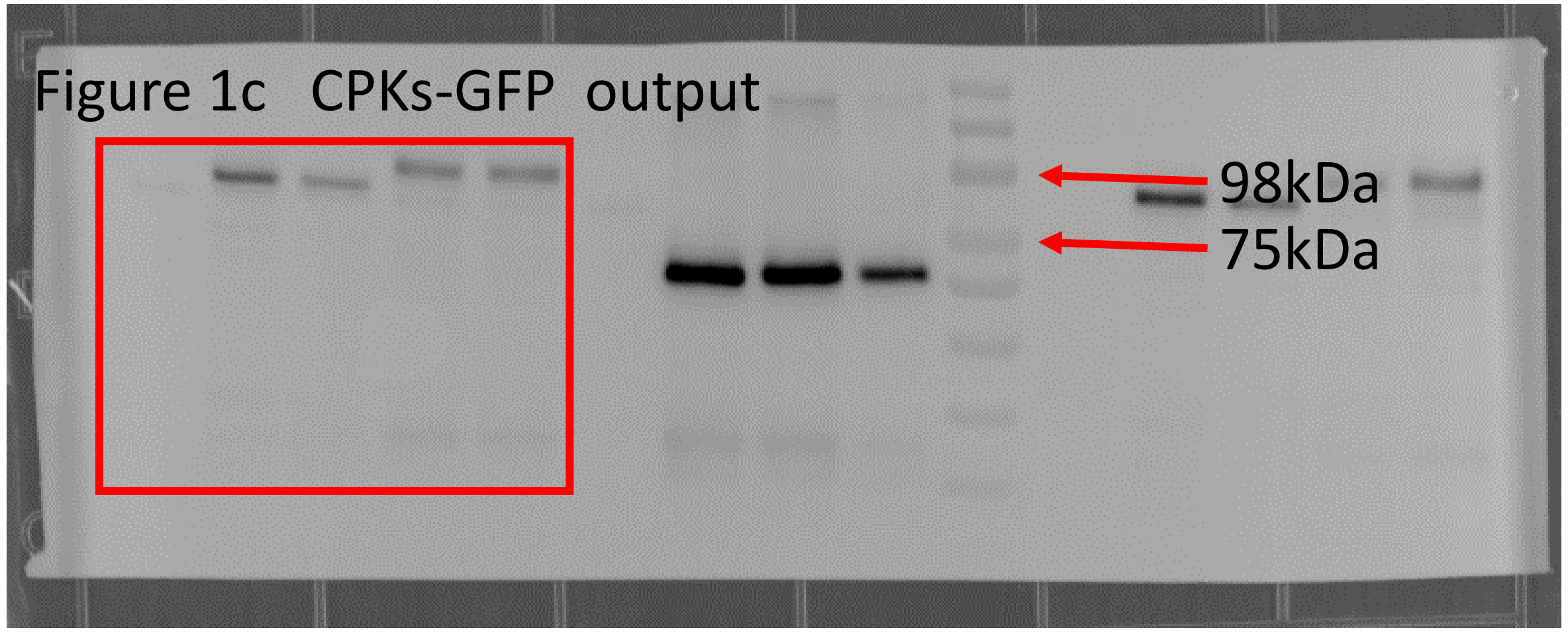
Figure 1c CPKs-GFP output
98kDa
75kDa

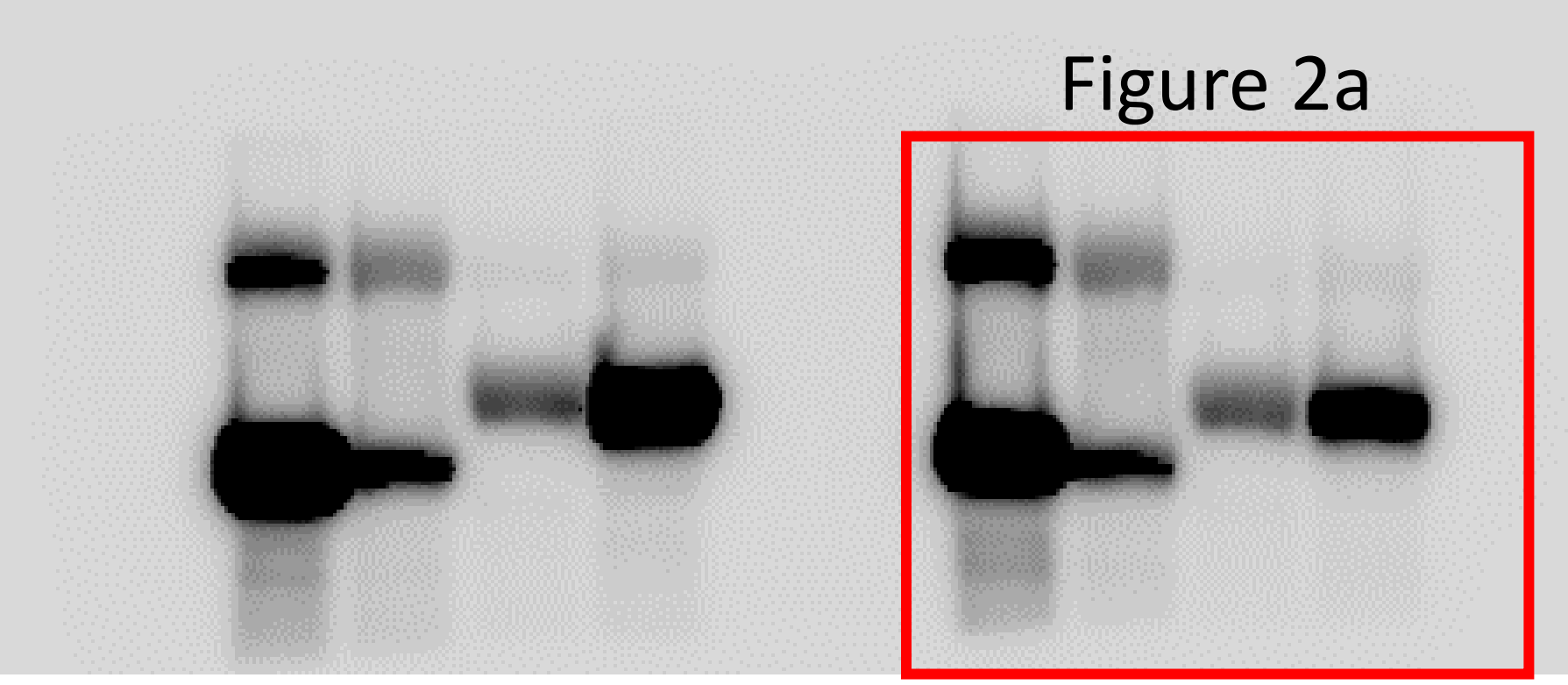

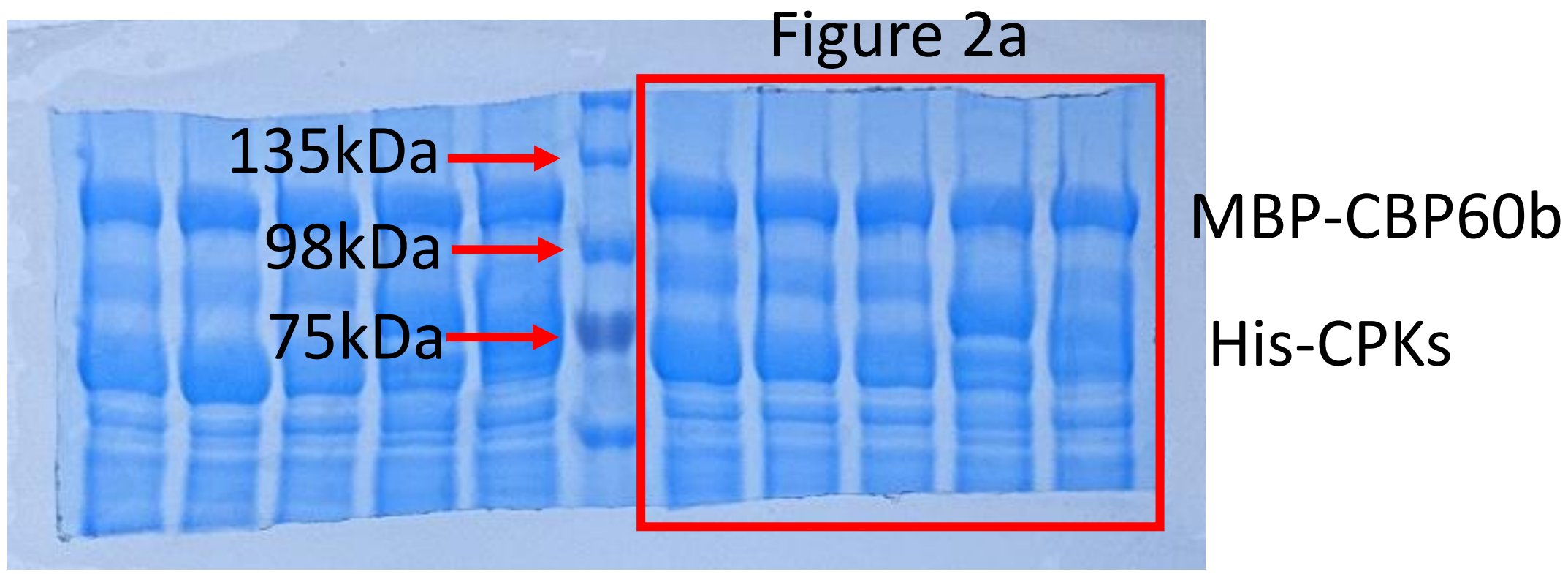

Figure 2b

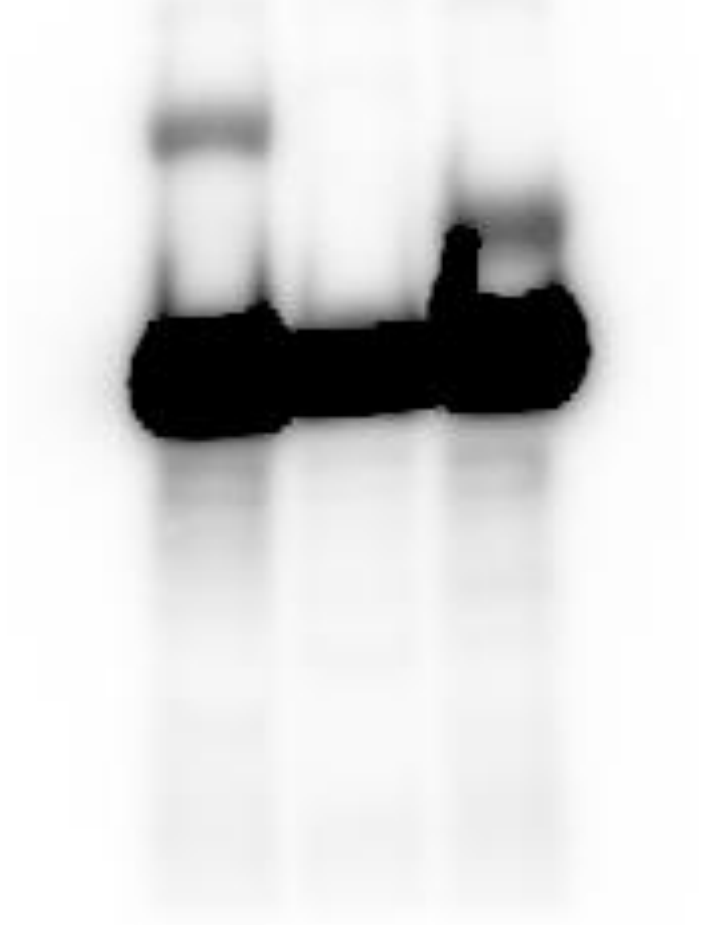

Figure 2b

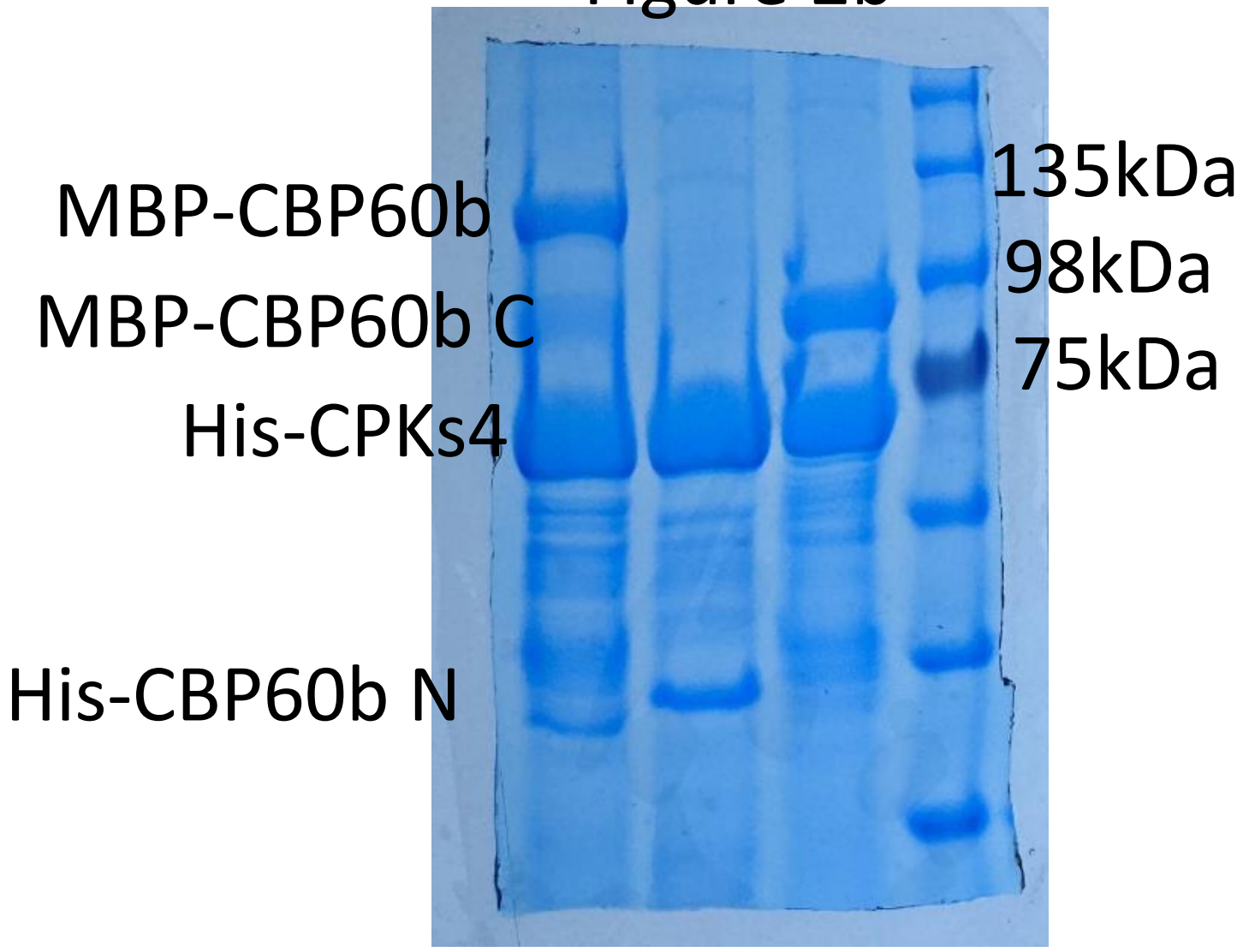

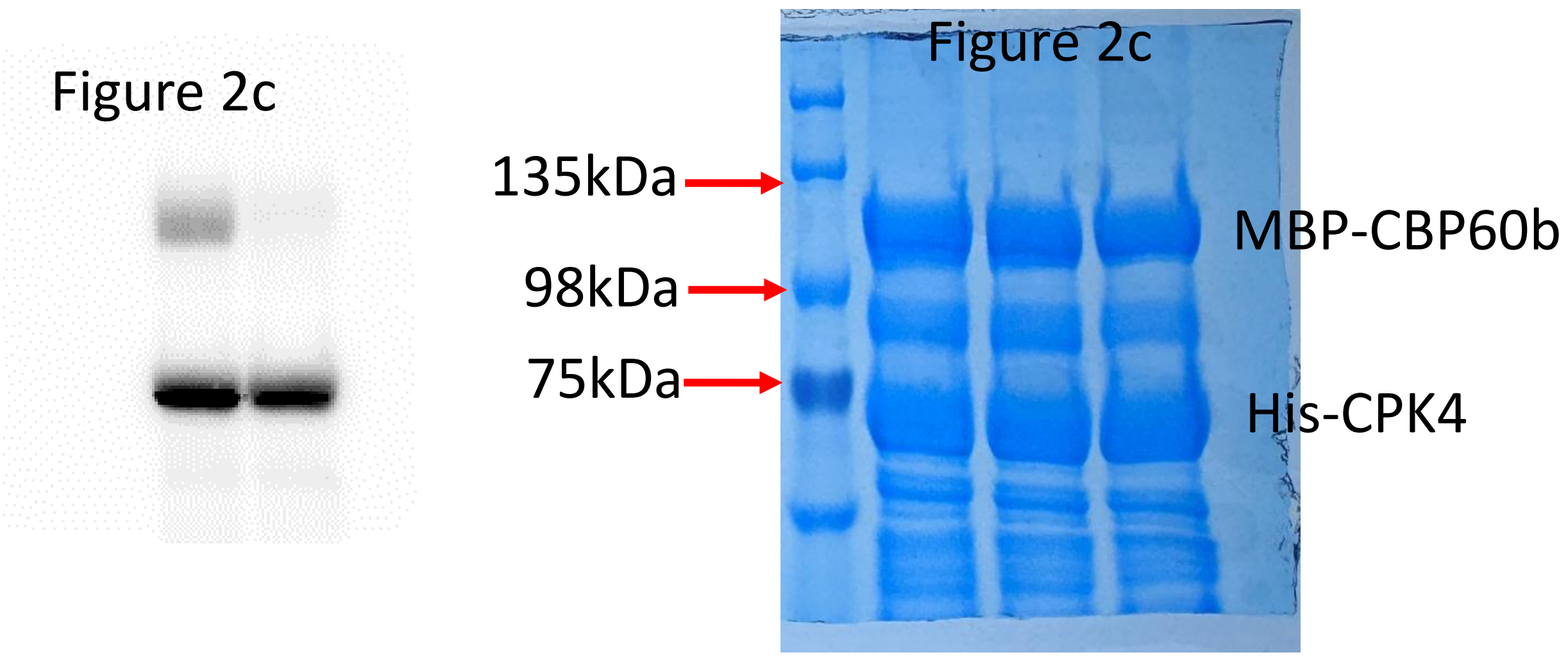
Figure 2c
Figure 2c
135kDa
98kDa
75kDa
MBP-CBP60b
His-CPK4

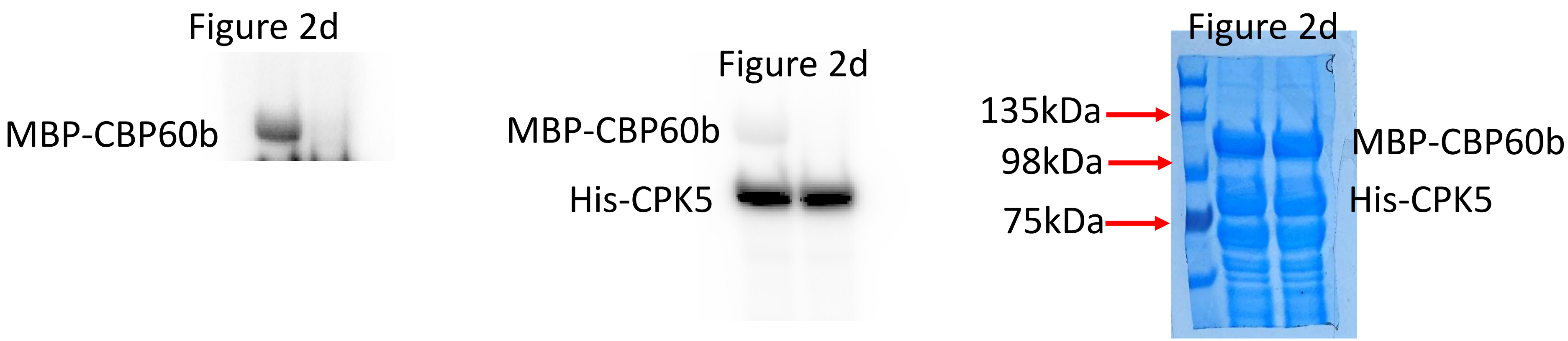
Figure 2d
MBP-CBP60b
Figure 2d
MBP-CBP60b
His-CPK5
Figure 2d
135kDa
98kDa
75kDa
MBP-CBP60b
His-CPK5

Figure S1A His-CPKs

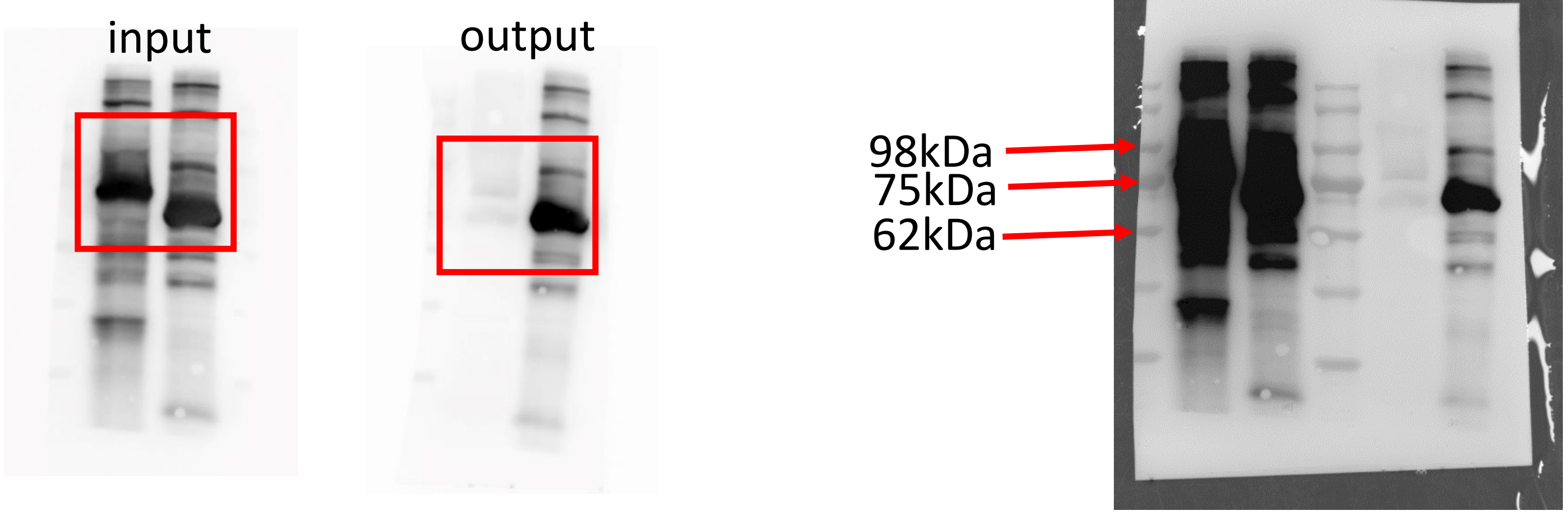

Figure S1A MBP-CBP60b

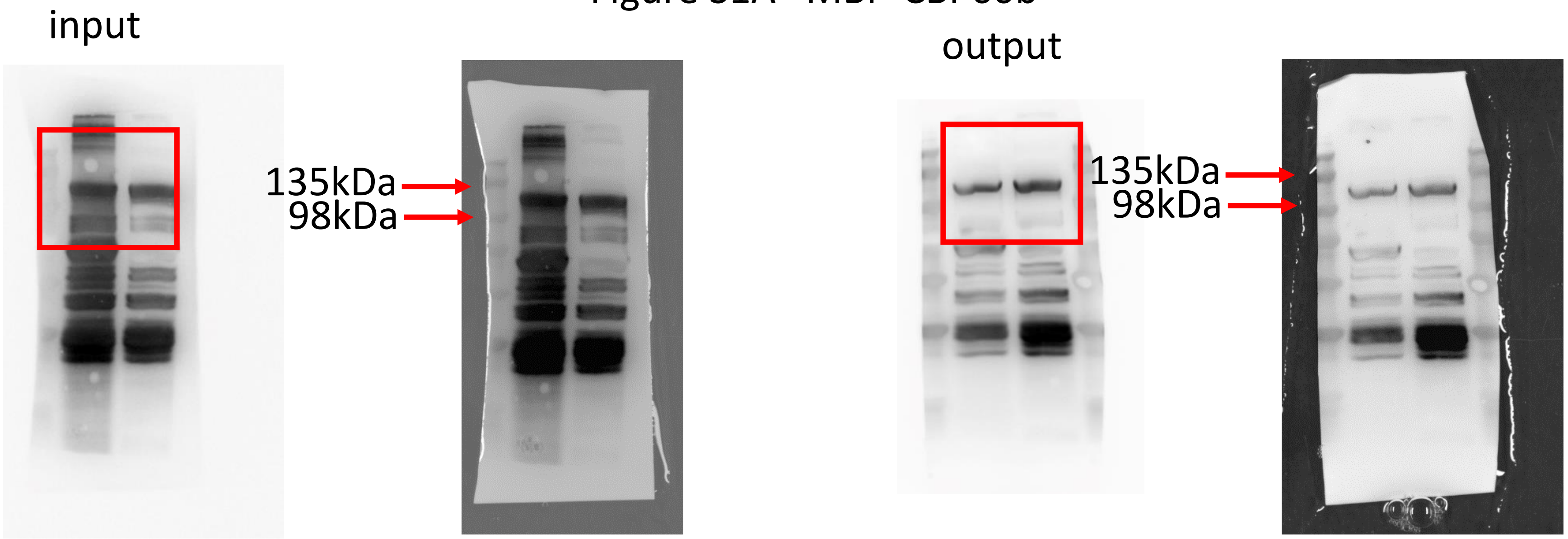

Figure S1B CPKs-GFP input

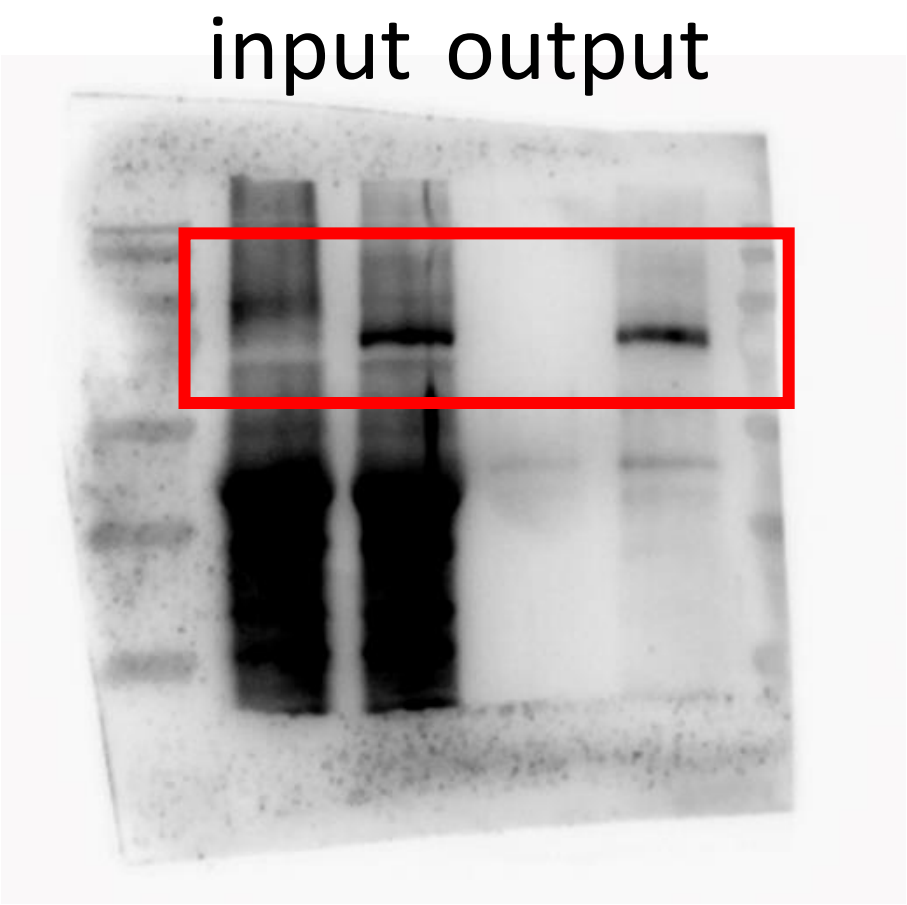

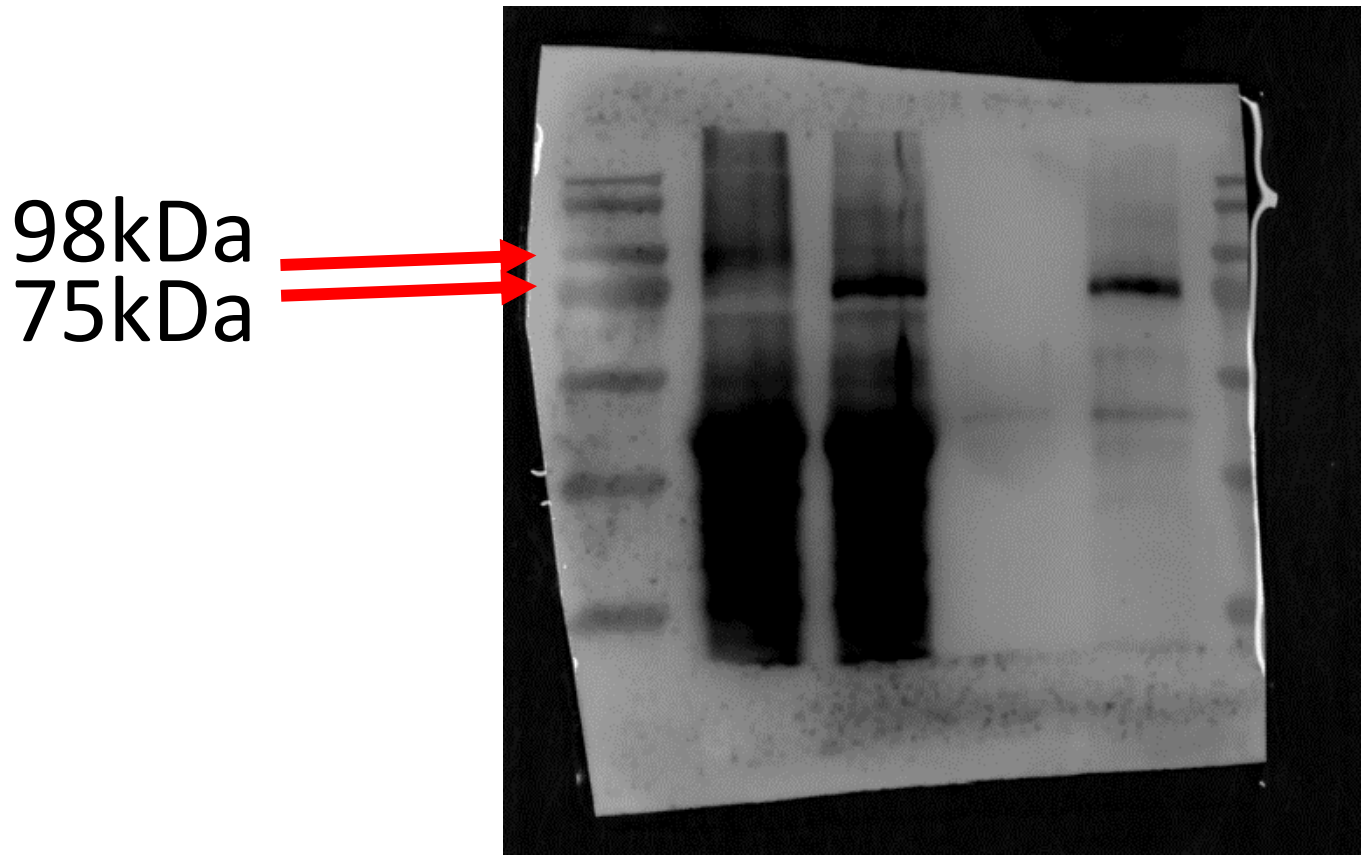

Figure S1B Flag-CBP60b

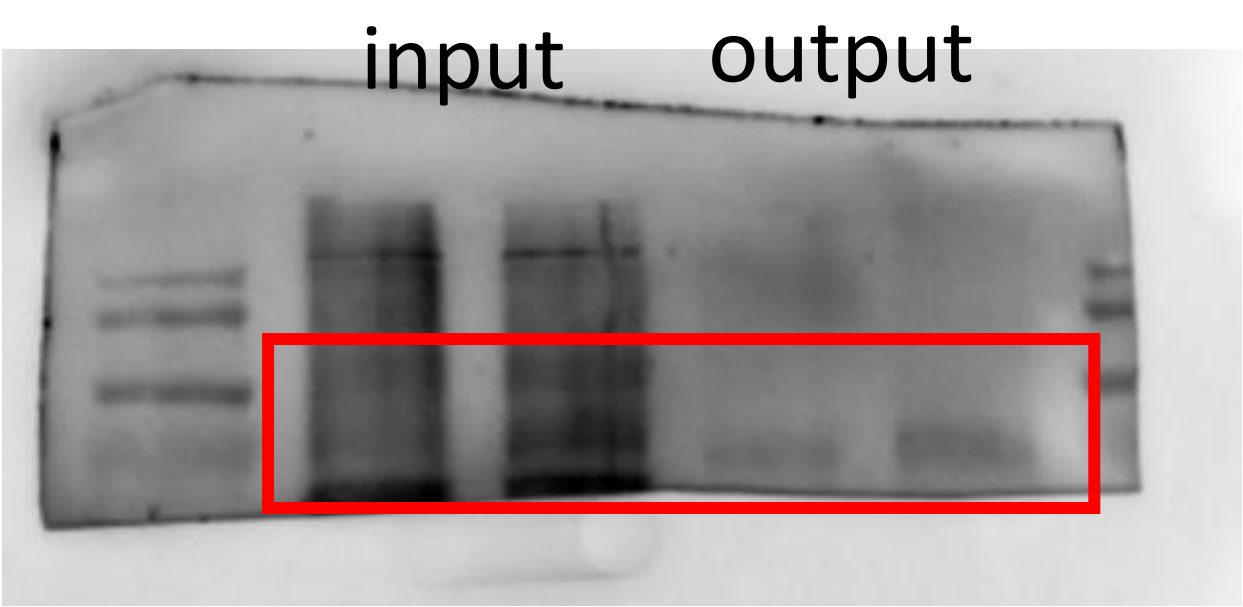

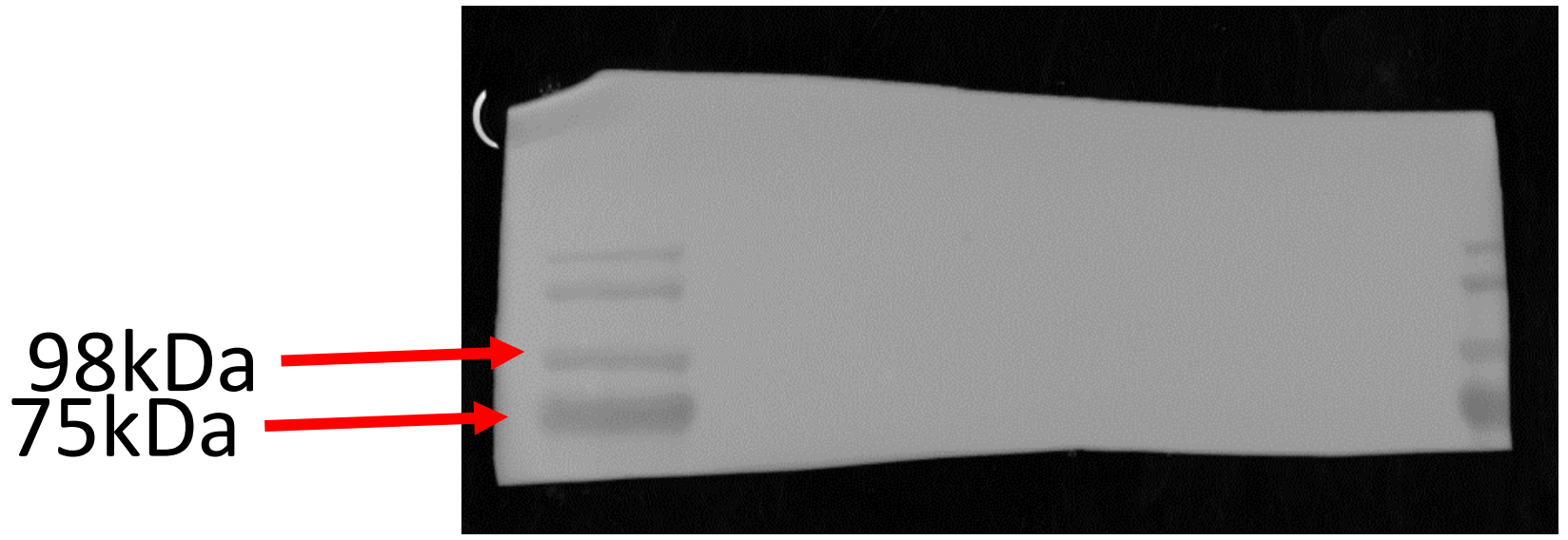

Figure S3A CBP60b-GFP

Phos-tag

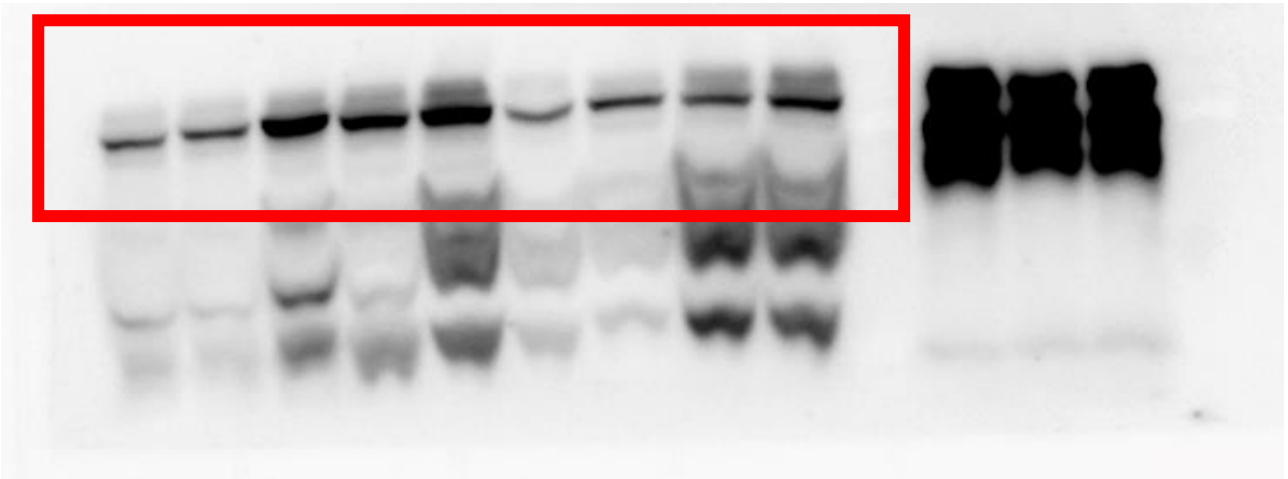

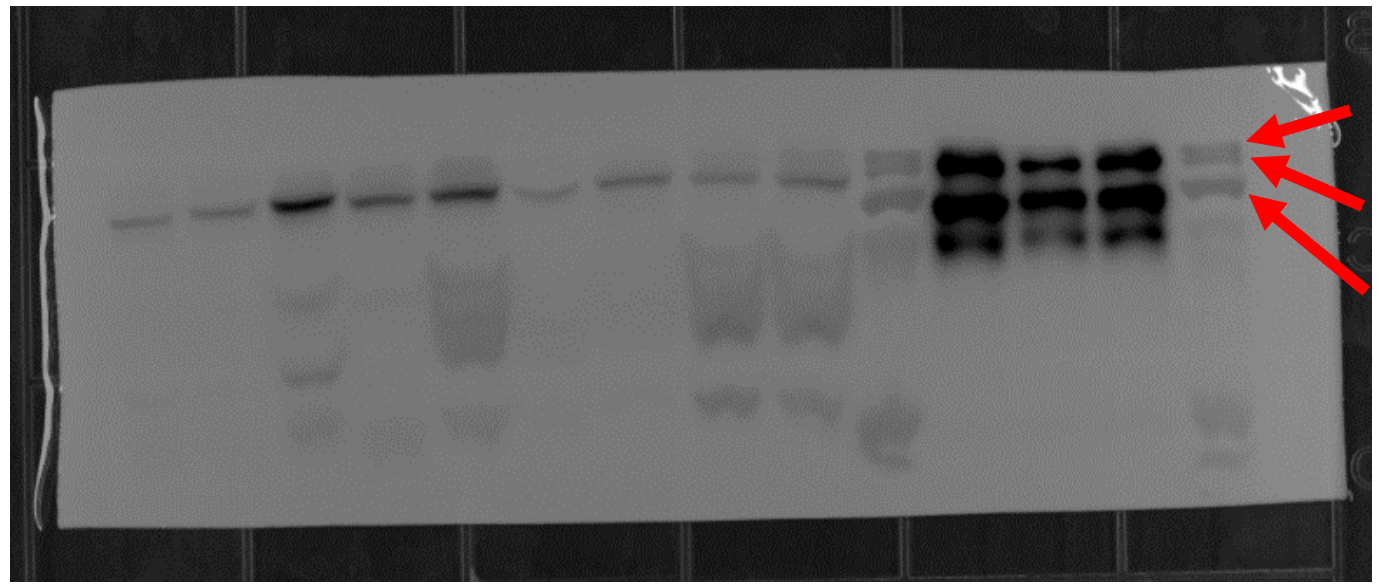

135kDa
98kDa
75kDa

Regular gel

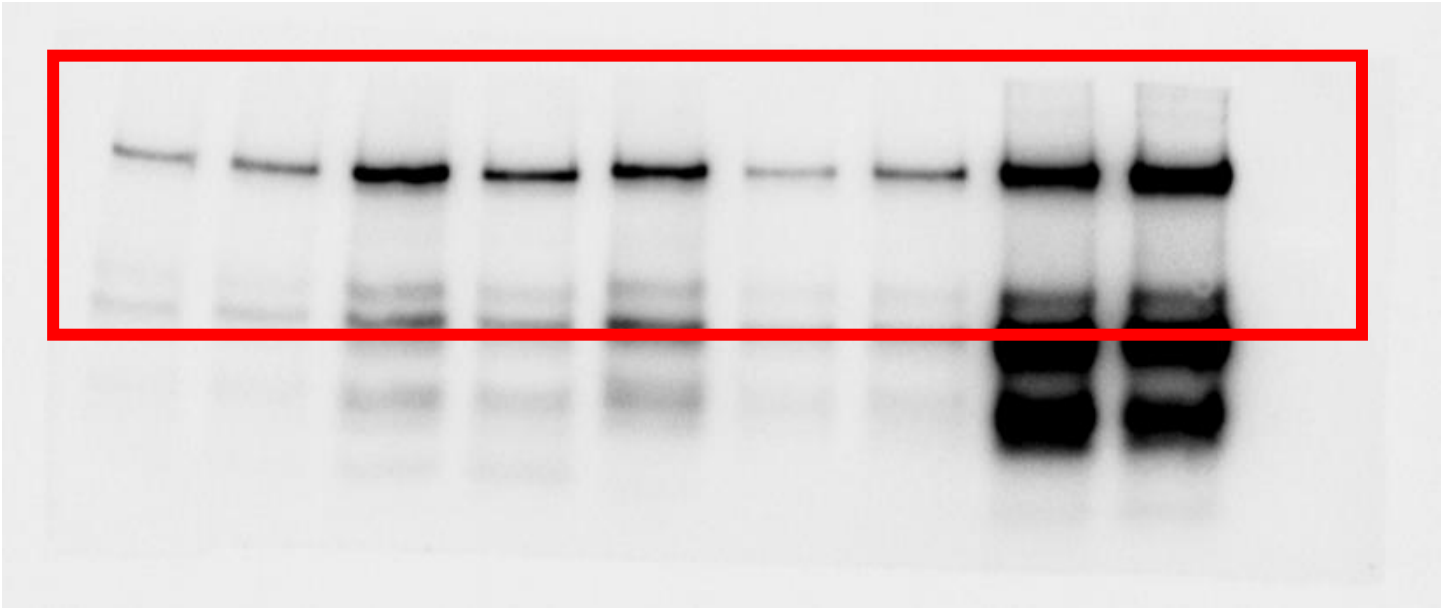

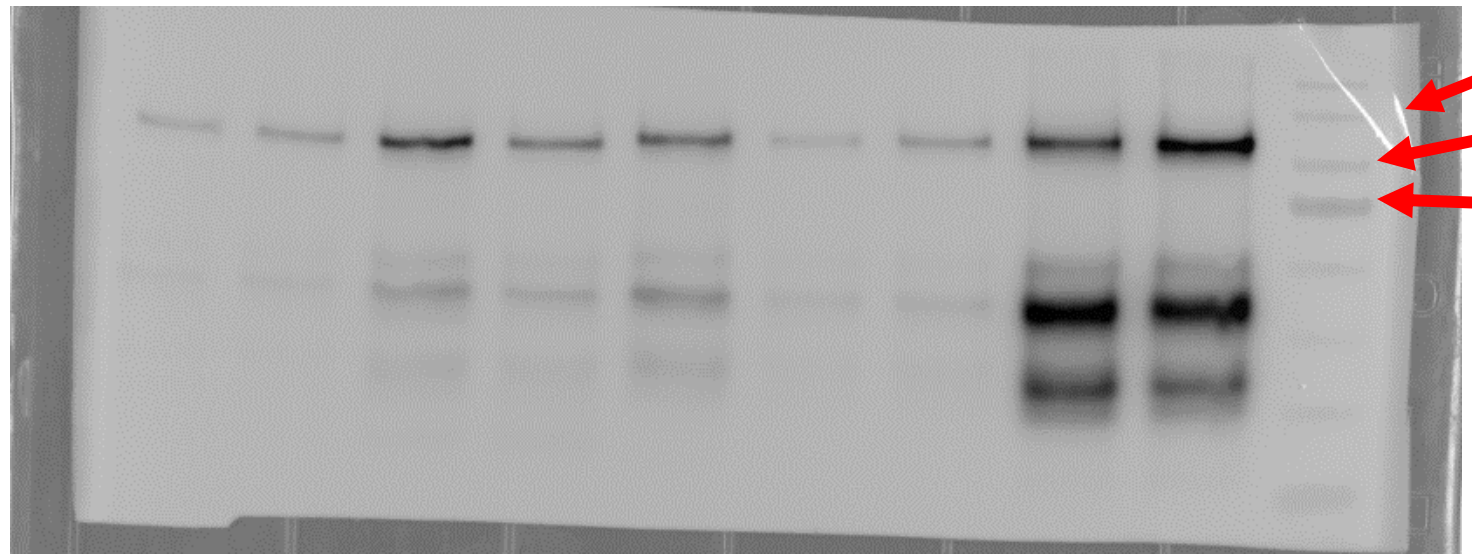

135kDa
98kDa
75kDa

Figure S3B CBP60b-GFP

Phos-tag

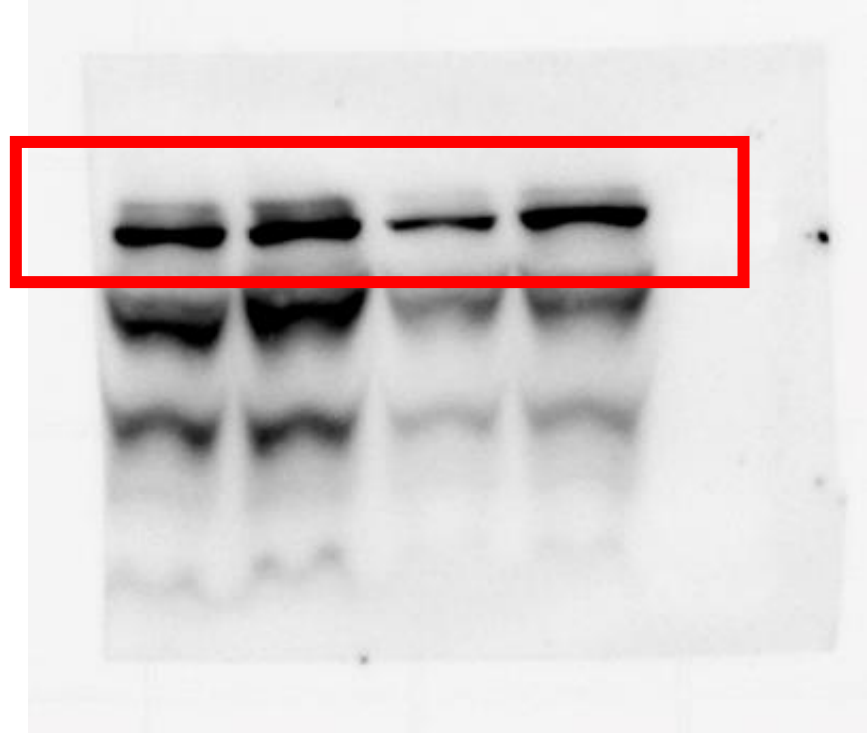

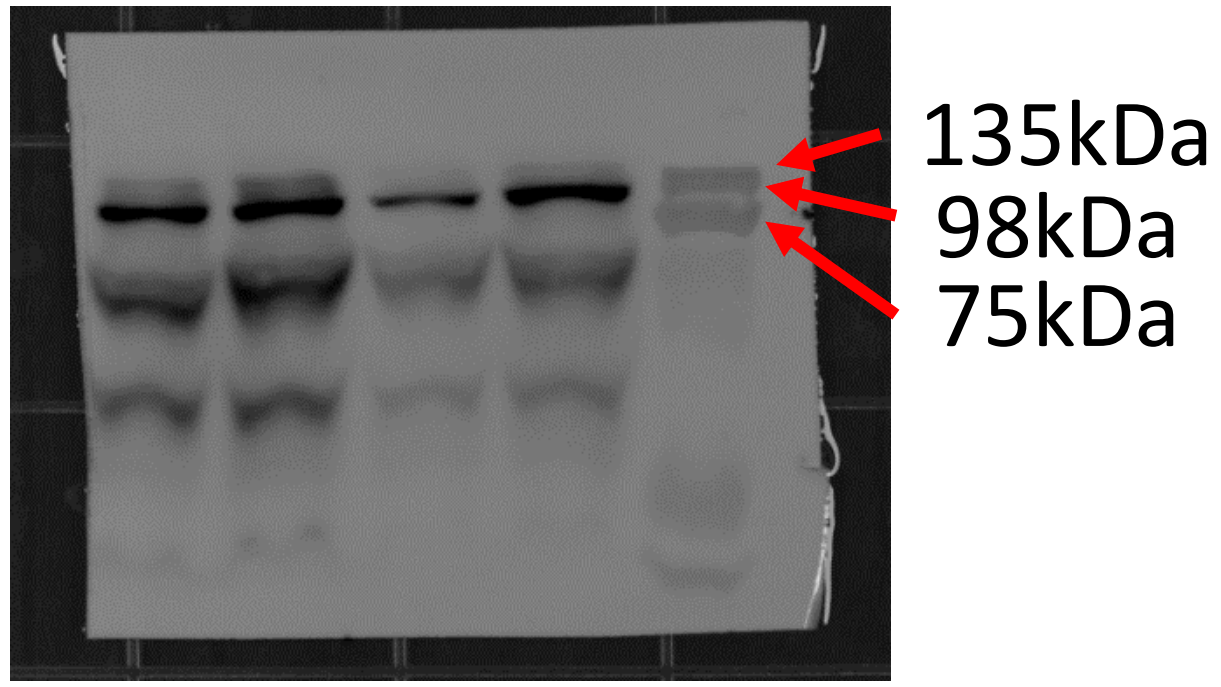

Regular gel

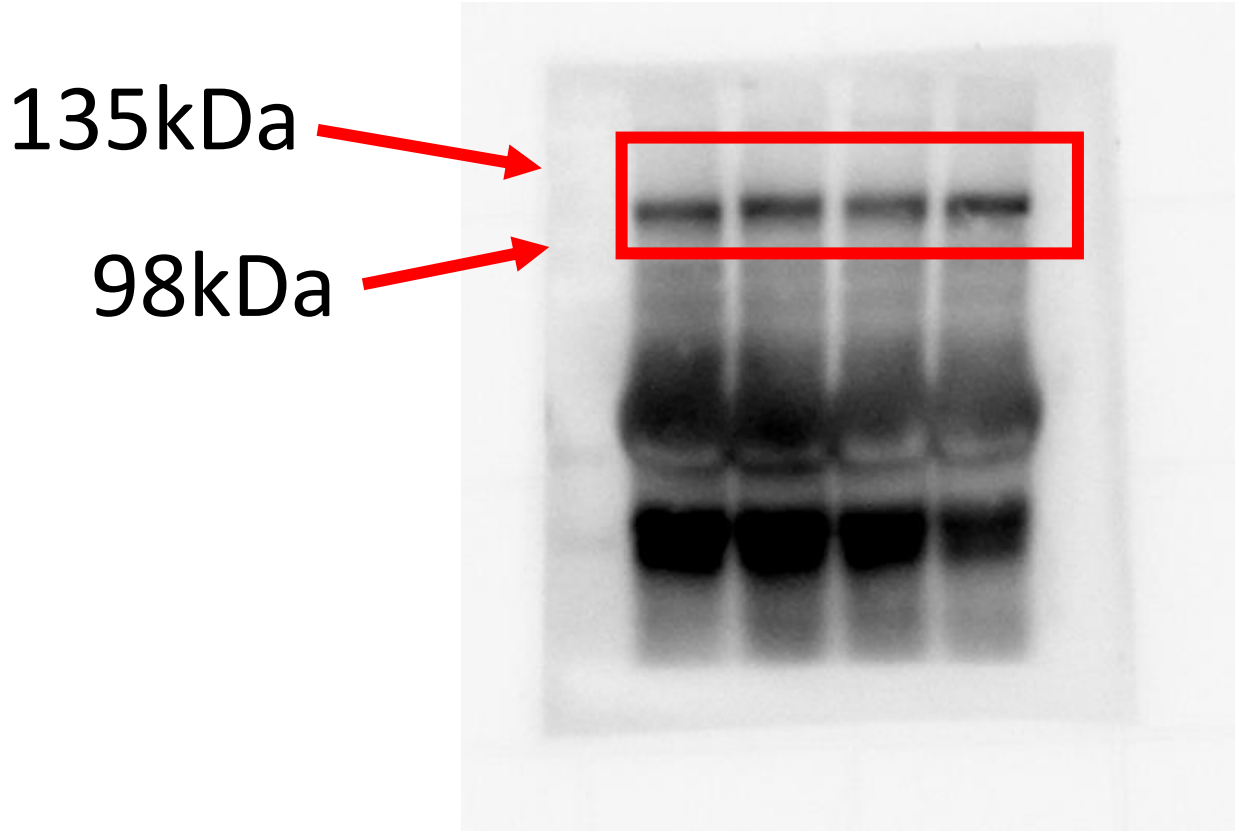

Figure S4C

CBP60b-GFP

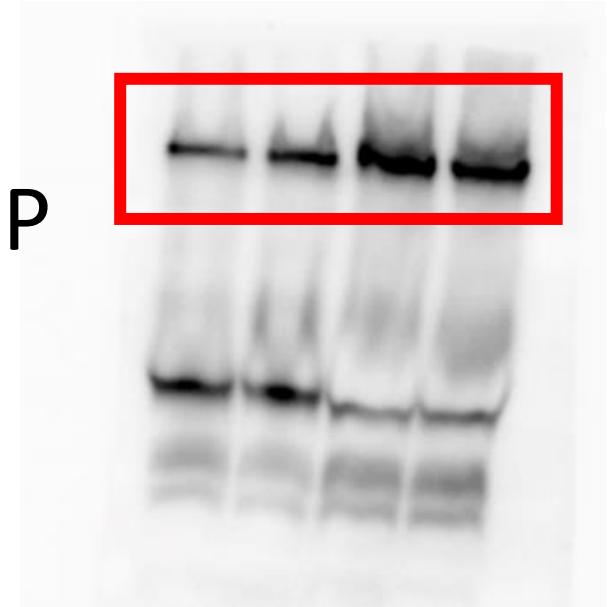

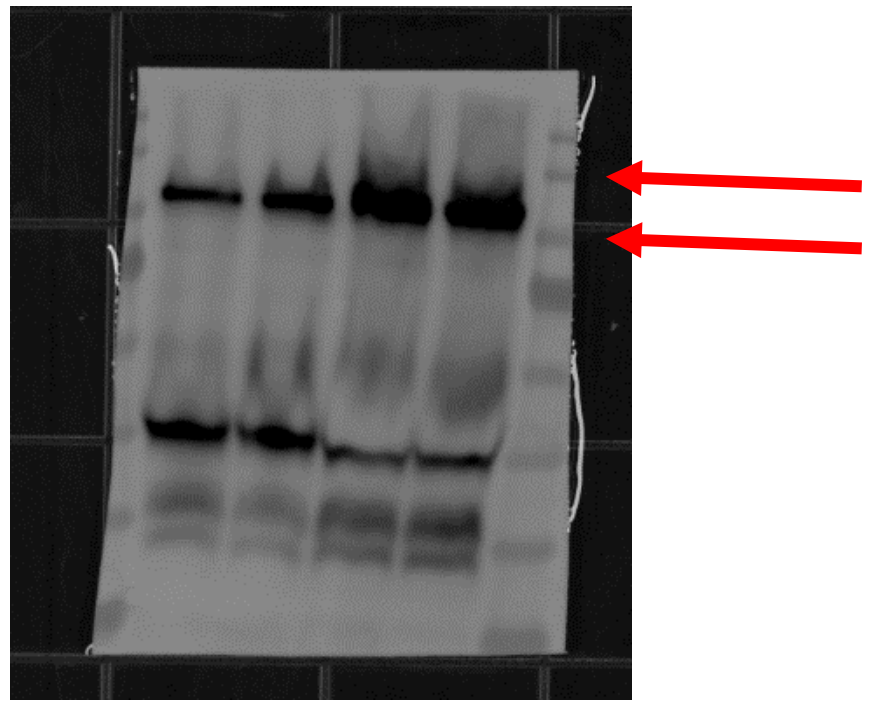

135kDa

98kDa

Actin

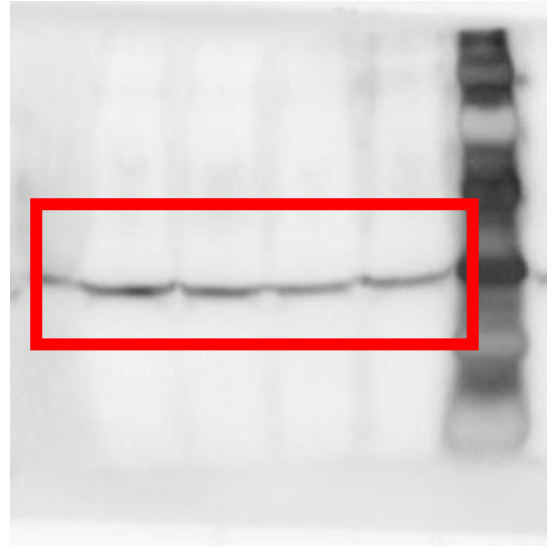

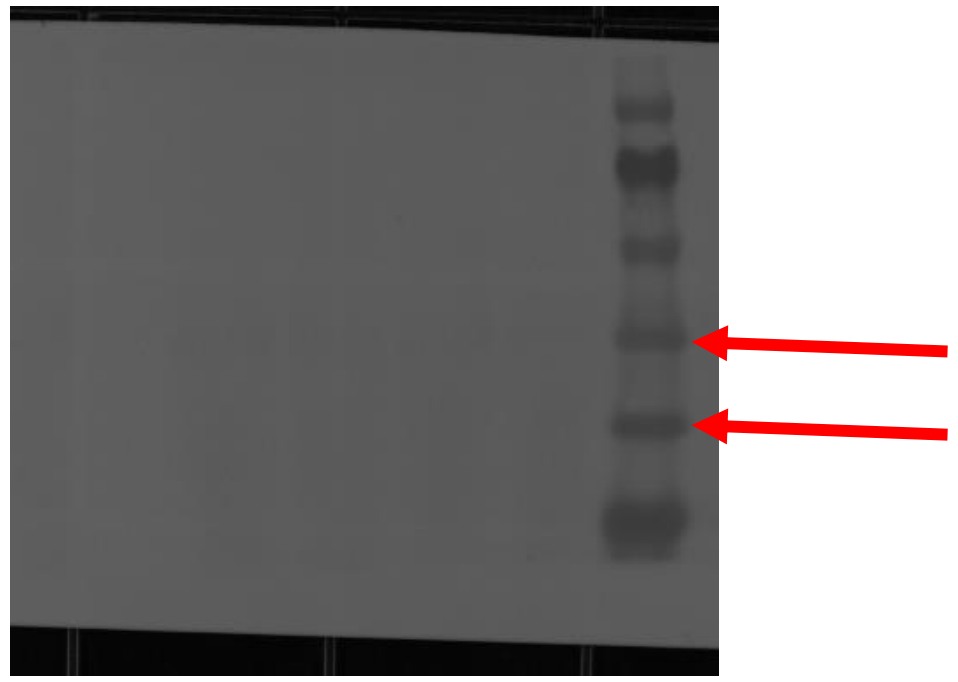

48kDa

34kDa

Figure S5

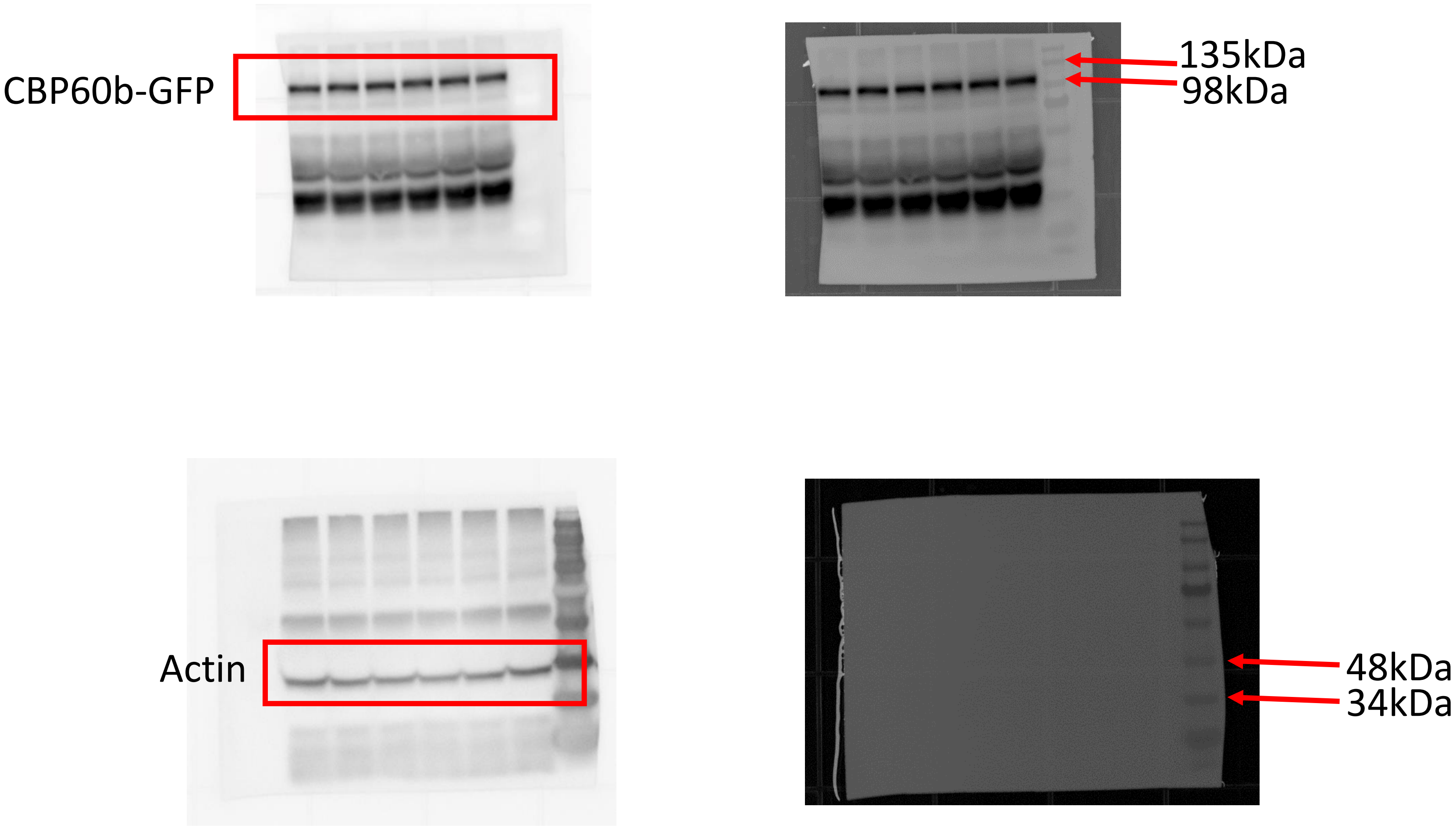

Supplement: Supplementary file 1 [file TPJ-127-0-s002.pdf]
