# Supplementary material for: The CPKs‐CBP60b positive feedback loop regulates immunity in Arabidopsis
Source: Plant J. 2026 Sep 8;127(5):e71100. doi: 10.1111/tpj.71100 (PMC13553119; doi:10.1111/tpj.71100)

Sequence: LFS568MGGGGNGSATHLPEDGYTFPSFLHTPMQGYDEDR, S3-Phospho (79.96633 Da)
Charge: +4, Monoisotopic m/z: 1017.93848 Da (+2.91 mmu/+2.86 ppm), MH+: 4068.73208 Da, RT: 56.5082 min,
Identified with: Sequest HT (v1.17); XCorr:7.90, Percolator q-Value:1.1e-3, Percolator PEP:2.3e-8,
Fragment match tolerance used for search: 0.02 Da

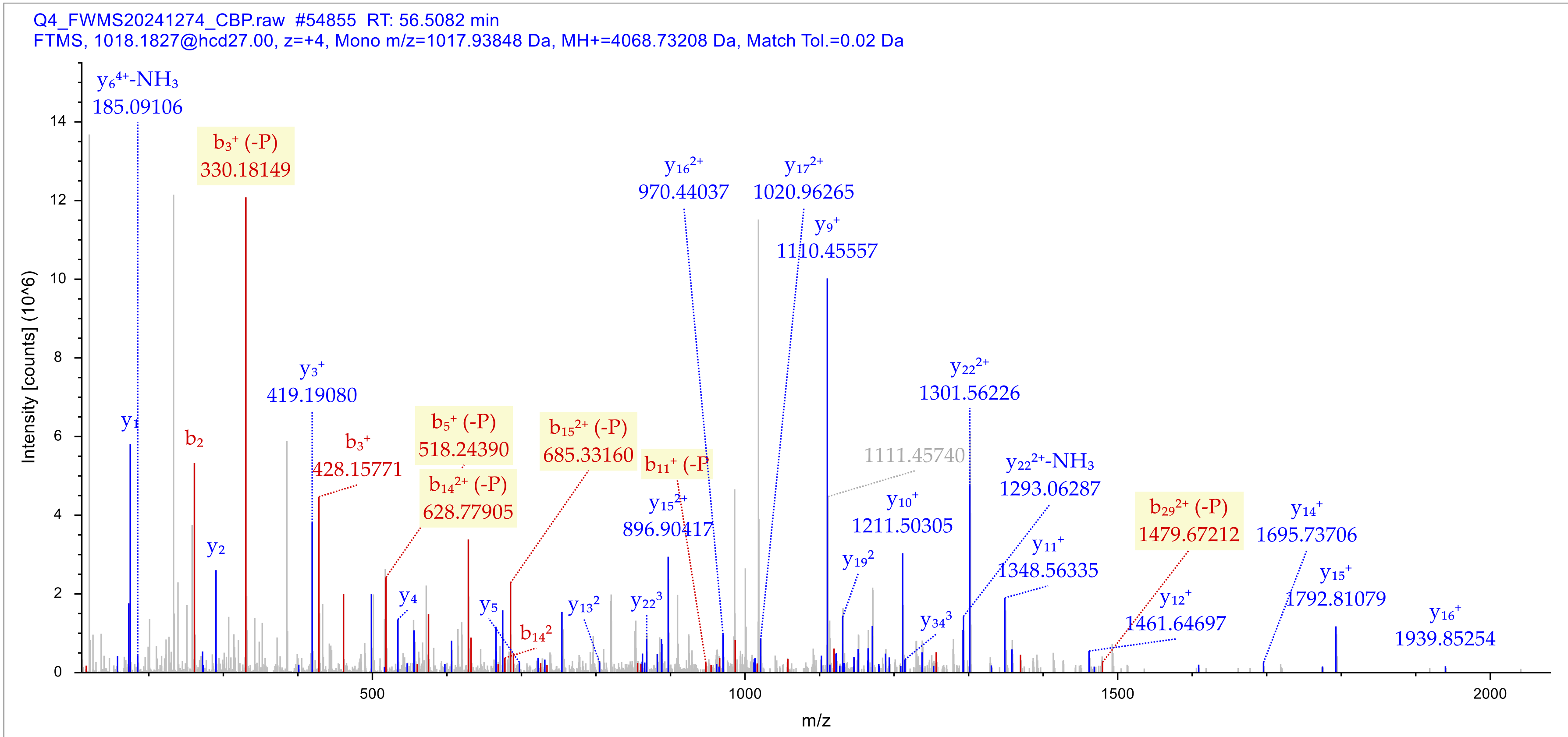

Supplement: Supplementary file 2 [file TPJ-127-0-s005.pdf]
