# Supplementary material for: The CPKs‐CBP60b positive feedback loop regulates immunity in Arabidopsis
Source: Plant J. 2026 Sep 8;127(5):e71100. doi: 10.1111/tpj.71100 (PMC13553119; doi:10.1111/tpj.71100)
Supplement: Supplementary file 3 [file TPJ-127-0-s001.docx]

**Supplemental Figure 1. The expression of immune genes induced by pathogens are reduced in *cpk4;5;6;11*.**

(A-D) Relative transcript abundance of *EDS1* (A), *SID2* (B), *SARD1* (C) and *CBP60g* (D) upon mock treatment (MgCl_2_) or *Pto* DC3000 treatment in wild type (WT) and *cpk4;5;6;11*. RNAs were extracted from infiltrated leaves at 24 h post-treatment. Results are means ± SE (n = 3). Asterisks indicate significant difference (*t* test; P < 0.05).

**Supplemental Figure 2. CBP60b interacts with CPK4 but not with CPK28.**

(A) Pull-down assays and (B) Co-IP assays showing that CBP60b interacts with CPK4 but not with CPK28.

**Supplemental Figure 3. Pathogen-induced phosphorylation of CBP60b is reduced in *cpk4;5;6;11*.**

(A-B) Two repeated experiments showing *Pto* DC3000-induced phosphorylation of CBP60b is impaired in the *cpk4;5;6;11* mutant. Five-week-old (5 WAG) WT and *cpk4;5;6;11* plants expressing CBP60b-GFP were infiltrated with *Pto* DC3000 (OD600 = 0.0001). Total proteins were harvested and separated by SDS-PAGE using gels with or without 30 mM Phos-tag and 60 mM MnCl2, and analyzed by anti-GFP immunoblotting. (C) Relative phosphorylation level in (A-B), Data are means (n = 2) with individual values shown as dots, calculated as the ratio of non-phosphorylated to phosphorylated band densities.

**Supplemental Figure 4. CPKs do not affect the localization and protein accumulation of CBP60b.**

(A-B) Representative CLSM of root meristematic zone cells from the CBP60b-GFP in WT (A) and in *cpk4;5;6;11* (B) at 5 DAG. FM4-64 staining was used to show the PM. Bars = 20 μm. (C) Relative protein abundance of CBP60b-GFP in WT or in *cpk4;5;6;11* by western blot analysis. Five-week-old (5 WAG) WT and *cpk4;5;6;11* plants expressing CBP60b-GFP were infiltrated with *Pto* DC3000 (OD600 = 0.0001). Total proteins were harvested 0 or 24 h post-infiltration. Anti-Actin was used as the internal loading control in the western blot assays.

**Supplemental Figure 5. The expression level of exogenous CBP60b is relatively consistent.**

Relative protein abundance of exogenous CBP60b in the 5 WAG indicated genotypes by western blot analysis. Anti-Actin was used as the internal loading control in the western blot assays.

**Supplemental Figure 6. CBP60b^S568A^ and CBP60b^S568D^ rescue the developmental defects of *cbp60b*.**

Representative growth of WT, *cbp60b-1* (*cbp60b*), two independent lines of *CBP60b^S568A^ genomic;cbp60b* and two independent lines of *CBP60b^S568D^ genomic;cbp60b* at 3 WAG under LD conditions.

**Supplemental Figure 7. CBP60g and SARD1 induce *CPK6* expression.**

Quantitative luminescence showing the LUC activity of *proCPK6:LUC* co-transfected with *35S:GFP* (GFP), *35S:CBP60g-GFP* (CBP60g) or *35S:SARD1-GFP* (SARD1). LUC reporter activity was determined 16 h post-transfection. Values are means ± SE (n = 3). Different letters indicate significantly different groups (1-way ANOVA and Tukey’s multiple comparison test; P < 0.05).
